# Biological Potential of Methanol Extracts from Plants of the Genus *Spiraea* Spreading in Russia

**DOI:** 10.3390/ijms26083587

**Published:** 2025-04-10

**Authors:** Anastasia Orlova, Alena Soboleva, Elena Tsvetkova, Svetlana Silinskaia, Yana L. Esaulkova, Tatiana N. Veklich, Vladimir V. Zarubaev, Anna A. Khakulova, Ilya R. Akberdin, Semyon K. Kolmykov, Vera A. Kostikova, Andrej Frolov

**Affiliations:** 1 Laboratory of Analytical Biochemistry and Biotechnology, K.A. Timiryazev Institute of Plant Physiology Russian Academy of Science, 35 Botanicheskaya Str., 127276 Moscow, Russia; oriselle@yandex.ru (A.S.); svetlanasilsv@mail.ru (S.S.); 2 Department of Biochemistry, St. Petersburg State University, 7-9-11 University Emb., 199034 St. Petersburg, Russia; e.v.tsvetkova@spbu.ru; 3 Department of General Pathology and Pathological Physiology, Institute of Experimental Medicine, 12 Acad. Pavlov Street, 197022 St. Petersburg, Russia; 4 The Laboratory of Experimental Virology, St. Petersburg Pasteur Institute, 14 Mira Str., 197101 St. Petersburg, Russia; esaulkova@pasteurorg.ru (Y.L.E.); zarubaev@pasteurorg.ru (V.V.Z.); 5 Botany Laboratory, Amur Branch of Botanical Garden-Institute of the Far Eastern Branch Russian Academy of Science (AB BGI FEB RAS), Ignatievskoe Road, 2km, 675000 Blagoveshchensk, Russia; tbliznjuk@mail.ru; 6 Chemical Analysis and Materials Research Core Facility Center, Research Park, Saint-Petersburg State University, 199034 St. Petersburg, Russia; a.khakulova@spbu.ru; 7 Department of Computational Biology, Sirius University, 1 Olimpiyskiy Avenue, 354340 Sochi, Russia; akberdin.ir@talantiuspeh.ru (I.R.A.); kolmykovsk@gmail.com (S.K.K.); 8 Laboratory of Phytochemistry, Central Siberian Botanical Garden of Siberian Branch of Russian Academy of Sciences (CSBG SB RAS), 101 Zolotodolinskaya Str., 630090 Novosibirsk, Russia; serebryakovava@mail.ru

**Keywords:** *Spiraea*, Rosaceae family, polyphenols, secondary metabolites, antioxidants, antimicrobial activity, antiviral activity

## Abstract

The genus *Spiraea* is well represented in the Russian flora. Several phytochemical and bioactivity studies, completed so far with several individual species of this genus, indicate young *Spiraea* shoots as a promising source of pharmaceutically and nutraceutically active natural products. Therefore, a broad-scale phytochemical analysis of shoot extracts from multiple Russian *Spiraea* species (i.e., profiling of secondary metabolites and assignment of their structures), complemented with comprehensive activity screening, might give access to valuable information on the structure–activity relationship (SAR) of their constituents. However, despite a lot of phytochemical and bioactivity information on individual species being available, these data are mostly fragmentary and do not allow for building a general picture, and in-depth comprehensive studies are still missing. Therefore, to fill this gap, here, we present a comprehensive metabolite profiling study accomplished with 15 of the most widely spread Russian *Spiraea* species, which was complemented with appropriate bioactivity screening of their first-year shoot alcoholic extracts. A chromatography–mass spectrometric (LC-MS) analysis revealed 33 major constituents of the shoot isolates, which were dominated by flavonoids (quercetin and kaempferol derivatives) and hydroxycinnamic acids (caffeic, ferulic, and coumaric acid derivatives). Their relative quantification indicated that most of the identified major components were distributed among all of the studied extracts with minimal overlap in their composition and relative abundance. The antioxidant activity screening revealed the high efficiency of all of the extracts as potential redox protectors, acting at the levels of radical scavenging (DPPH assay) and quenching cation radicals (TEAC assay) and superoxide anion radicals (NBT assay). Screening the antiviral and antimicrobial activity of the same extracts revealed significant antiviral activity at a concentration of 2 µg/mL, and high (MIC < 1 mg/mL) or moderate (1 mg/mL ≤ MIC ≤ 4 mg/mL) antibacterial activity against Gram-positive and Gram-negative strains. The structures responsible for the manifestation of the studied types of activity were tentatively assigned using a bioinformatics-based strategy. This analysis revealed the most bioactive *Spiraea* species that might be promising for further in-depth phytochemical analysis and evaluations of their structure–activity relationships (SARs). In this context, we consider *S. humilis*, which simultaneously showed antioxidant, antimicrobial, and antiviral activity; *S. media*, with marked antioxidant, antimicrobial, and cytotoxic properties; *S. ussuriensis*, a strong antioxidant and cytotoxic species; and *S. trilobata*, with a combination of antioxidant and antiviral properties.

## 1. Introduction

The potential biological activity of medicinal plants relies on so-called biologically active complexes (BACs)—compounds that can have a pronounced effect on life processes at low concentrations [1]. Upon consumption by animals or humans, the constituents of these complexes are absorbed in the gastrointestinal tract and transported to the target organs and cells to exert their therapeutic effect [2]. Knowledge about the composition of naturally occurring BACs, and the structures, properties, and activity profiles of their constituents might give access to prototypes of new prospective highly efficient drugs. To gain this valuable information, in-depth analyses of the chemical diversity of plants with well-characterized biological activity are universally recognized as an efficient strategy for discovering promising drug prototypes [3]. The other option is the comprehensive and systematic evaluation of taxons containing species with confirmed biological activity. On one hand, new plants with unknown bioactivity profiles might have a chemical composition similar to that of their well-characterized relatives, which facilitates phytochemical studies. On the other, due to intra-taxon biochemical diversity, such valuable chemical information might essentially widen the scope of the drug candidate search [4].

The genus *Spiraea* (*Spiraea*) represents one such understudied taxon. It belongs to the tribe *Spiraeeae* of the subfamily *Amygdaloideae* (family *Rosaceae*) and includes about 90 species which inhabit the temperate zone of the Northern Hemisphere. In Russia, the genus *Spiraea* is represented by 20–25 species, which are distributed in four sections: *Spiraea* Ser., *Calospira* K. Koch, *Chamaedryon* Ser., and *Glomerati* Nakai. Remarkably, these species are unevenly distributed throughout the territory of the country. Thus, most of the Russian *Spiraea* representatives (20–23) are found in the Asian part of Russia [5,6,7,8], whereas only 2–3 species have been described in the European part of Russia [9].

The leaves of this genus are petiolate, simple, without leaflets, narrow-lanceolate to rounded, entire, 3-5-lobed, and simple or double-toothed. The inflorescences of spring-flowering species are sessile or almost sessile umbrellas, or shield-shaped brushes with a rosette of leaves or scales at the base. The species flowering in June and July have simple or compound corymbs at the ends of the generative short, mostly leafy twigs or at the ends of the shoots of that year. Finally, the species flowering in July and August have narrow-cylindrical, broadly pyramidal or elliptical panicles developing at the ends of the long, leafy shoots of that year. Their flowers are oviparous, white, light or dark pink, or red to purple, and appear from April to October [10,11]. The fruits of *Spiraea* are multi-seeded leaflets opening along the inner and later along the outer sutures. The seeds of *Spiraea* species are flat, lanceolate, brown, 1.5–2 mm long, and 0.5 mm wide.

*Spiraea* are widely used all over the world as ornamental shrubs for the landscaping of cities and settlements, as fodder, and as melliferous and soil-strengthening plants [7]. However, the species of the genus *Spiraea* are also of considerable interest as plants used in folk medicine. Thereby, their impressing resource potential represents the principal advantage of spiraeas as a source of biologically active natural products. Various species of the genus *Spiraea* are used in folk medicine in Asian countries for the treatment of malaria and as anti-inflammatory agents [12,13,14]. In traditional Chinese medicine, young leaves, fruits, and roots of *S. japonica* L. and its varieties are used as diuretics and analgesics [15,16]. Decoctions and infusions of *S. salicifolia* L. are used for the treatment of gastrointestinal diseases, rheumatism, helminthiasis, gynecological diseases, and diabetes [17,18]. The extracts obtained from the plants of the genus *Spiraea* show antioxidant [19,20], antimicrobial [21,22], antitumor [23,24], anti-inflammatory [25,26], and other types of biological activity.

According to data from the literature, several classes of biologically active natural products (i.e., secondary compounds with high biological activity) have been identified in *Spiraea* plants. The most common of the presented classes of compounds are phenolic compounds, among which are representatives of the flavonoids group, including derivatives of quercetin, kaempferol, apigenin, luteolin, and catechin, as well as derivatives of phenolic acids, among which the most common are derivatives of caffeic acid and cinnamic acid, as well as derivatives of syringic, coumaric, chlorogenic, gallic, and other acids [27,28,29,30,31]. In addition, the content of stilbenes such as 3-*O*-β-*D*-glucoside-5,4′-dihydroxystilbene and 3,5,4′-trihydroxystilbene in *S. formosana* extracts or resveratrol in *S. trilobata* leaf extracts has been reported in some species of the genus *Spiraea*. In studies of *S. japonica* var. *ovalifolia*, *S. prunifolia* var. *simpliciflora*, and *S. pubescens*, a number of compounds of the lignans group have been identified [32,33]. Compounds with a terpenoid structure, including derivatives of lupeol, oleanolic acid, betulinic acid, ursolic acid, and tormentic acid, have previously been found in *S. formosana*, *S. salicifolia*, *S. japonica* var. *ovalifoliab*, and *S. pubescens*, and some other species [27,34,35]. It was shown that the accumulation of diterpene alkaloids is characteristic of some species of the genus *Spiraea* growing in Southeast Asia [28], as well as the composition of components of fatty and essential oils of some species [29].

To date, only limited information has been reported about the constituents of the spiraeas growing in Russia. Thus, the chemical composition of the most widespread *Spiraea* species—*S. salicifolia* and *S. media* Franz Schmidt—has been most comprehensively studied, whereas that of the other Russian species has been poorly studied in respect of their secondary metabolite profiles and associated biological activities. The biological activities of such *Spiraea* species as *S. humilis*, *S. elegans*, *S. sericea*, *S. trilobata*, and *S. pubescens* were only minimally addressed in this respect, most likely due to their limited occurrence [28]. Therefore, the aim of this work was a comparative phytochemical study of methanol extracts from shoots of the first year of 15 taxa of the genus *Spiraea* growing in Russia.

## 2. Results

### 2.1. Profiling of Secondary Metabolites in the Methanolic First-Year Shoot Extracts of Spiraea spp.

To date, studies of the biological activity of plant isolates have ultimately used at least a qualitative analysis of the plant secondary metabolome. The identification and unambiguous structural characterization of potential bioactives is universally recognized as the first step in the primary work on the development of promising pharmaceutical agents in terms of evidence-based medicine. Over the last few decades, medicinal plants have been considered not only as sources of preparations based on crude extracts, but also as potential producers of highly biologically active purified individual natural products and their complexes.

**Figure 1:**
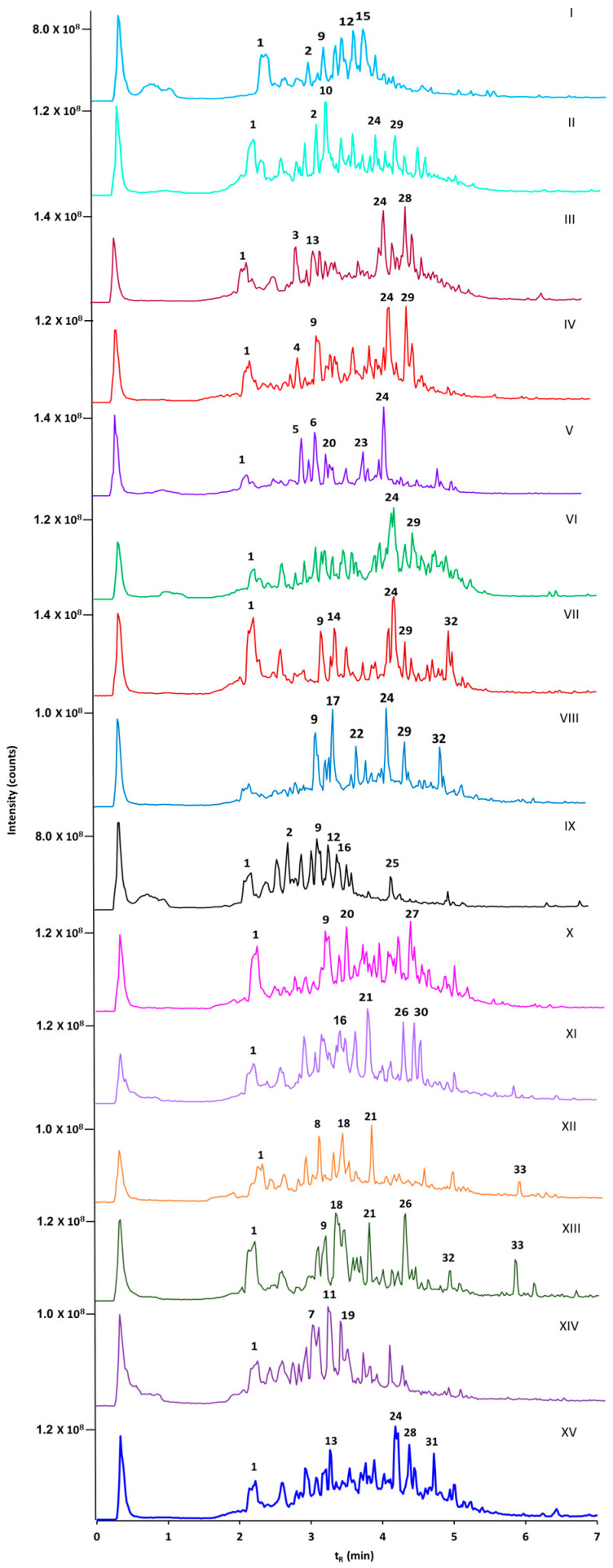
Total ion current chromatograms (TICs) of methanol extracts obtained from the first-year shoots of 15 species of the genus *Spireae* by reversed-phase ultra-high-performance quadrupole time-of-flight chromatography–mass spectrometry with electrospray ionization (RP-UHPLC-QqTOF-MS). The analyzed species of the genus *Spireae* are labeled as follows: I—*S. media*; II—*S. betulifolia*; III—*S. salicifolia f. alpestris*; IV—*S. pubescens*; V—*S. humilis*; VI—*S. flexuosa*; VII—*S. hypericifolia*; VIII—*S. aquilegifolia*; IX—*S. sericea;* X—*S. trilobata*; XI—*S. ussuriensis*; XII—*S. chamaedryfolia*; XIII—*S. crenata*; XIV—*S. elegans*; XV—*S. salicifolia*. Numbers 1–33 denote the semi-polar secondary metabolites, which were assigned as the major constituents of the extracts according to Table 1.

That is why, to address the pharmacological potential of the fifteen seemingly most widely occurring *Spiraea* species in Russia, we performed comparative metabolomics profiling of the corresponding methanolic extracts of their first-year shoots. Realizing that, most likely, the biological activities of the extracts are defined by the most abundant metabolites, we focused on the most well-represented semi-polar secondary metabolites yielding the most intense signals even in total ion current chromatograms (TICs), acquired in corresponding RP-UHPLC-QqTOF-MS/MS experiments (Figure 1).

Based on these considerations and having in mind limitations of the conventional phytochemical approach (which is absolutely compulsory for plant-derived drug establishment at more advanced stages), we considered approximately only two dozen of the most intense signals, i.e., those exceeding 4.5 ^ 10^7^ counts in the total ion chromatograms (TICs). The selected signals above this threshold were at least five-fold more intense than all others, and highly likely represented the compounds underlying pharmacological activity [30].

The interpretation of all of the TICs across the whole dataset in this way revealed, in total, 33 major semi-polar metabolites, which could be retained on the reversed phase. Among these, the annotation of 17 metabolites relied on the SWATH-MS data (Supplementary Materials, Figure S1), whereas the structural characterization of the remaining 16 compounds required additional tandem mass spectrometric (MS/MS) experiments (Supplementary Materials, Figure S2). The detailed related chromatographic and spectral information is summarized in Table 1.

The preliminary annotation of the major semi-polar constituents of the 15 prepared methanol extracts showed that polyphenols represented the main group of the principal extract components. Thereby, most of the annotated polyphenols were represented by flavonoids (which were dominated by the derivatives of quercetin and kaempferol) and hydroxycinnamic acids (caffeic and coumaric acid derivatives).

Among the annotated compounds, twelve compounds featured a common fragmentation pattern, dominated by signals at *m*/*z* 300.0281 characteristic for quercetin derivatives [30] or signals at *m*/*z* 301.0346, which correspond to the quercetin aglycon fragment. Among the compounds belonging to this group were compounds **2**, **4–6**, **8–9,** and **12–18**. The chromatographic and spectral information of these compounds is represented in Table 1 and their patterns of fragmentation are represented in the Supplementary Materials, Figures S1-2–S1-7, S1-9–S1-12, S2-2, and S2-4–S2-6. The comprehensive description of these data is provided in the Supplementary Information.

**Figure 2:**
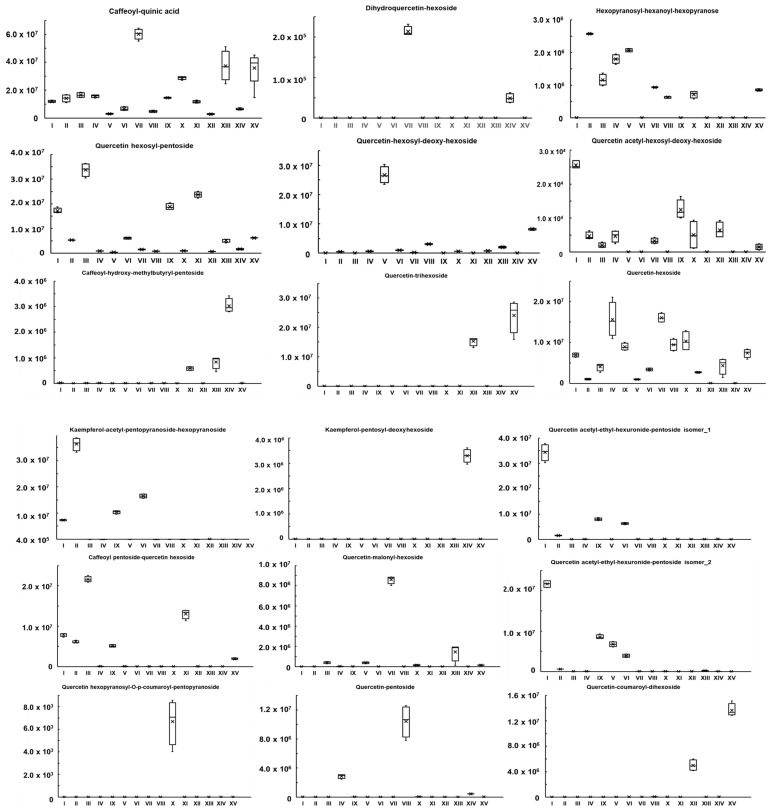

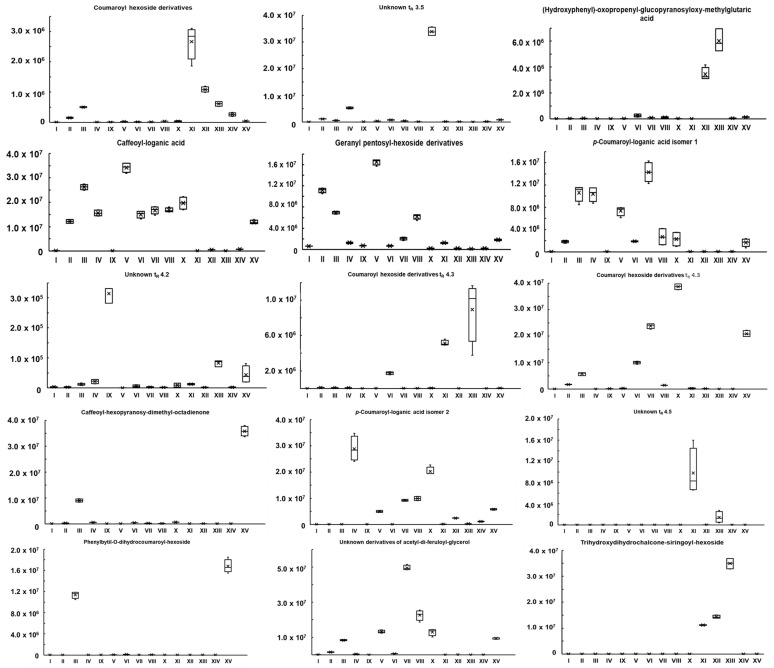
Relative recoveries of the major components in methanol extracts of first-year shoots of 15 plant species of the genus *Spiraea*, expressed as boxplots. Major components are named according to Table 1. The analyzed species of the genus *Spireae* are labeled as follows: I—*S. media*; II—*S. betulifolia*; III—*S. salicifolia f. alpestris*; IV—*S. pubescens*; V—*S. humilis*; VI—*S. flexuosa*; VII—*S. hypericifolia*; VIII—*S. aquilegifolia*; IX—*S. sericea;* X—*S. trilobata*; XI—*S. ussuriensis*; XII—*S. chamaedryfolia*; XIII—*S. crenata*; XIV—*S. elegans*; XV—*S. salicifolia*.

The relative recoveries of the major components in the investigated methanol extracts of the first-year shoots prepared from the 15 plant species from the genus *Spiraea* were identified using extracted ion chromatograms for each of the major components considered. The results of the relative recoveries of the components are presented in Figure 2.

### 2.2. Antioxidant Effects of the Methanolic Shoot Extracts of Spiraea spp.

The antioxidant effects of the *Spiraea* shoot extracts were addressed by three independent tests. Thus, the DPPH (2,2-diphenyl-1-picrylhydrazyl) assay showed that the shoot extracts of *S. humilis* appeared to be the strongest antioxidants with statistically significant differences from those of the other species of the studied genus (99.4% normalized for ascorbic acid, *p* < 0.01, Table 2), whereas the extracts of *S. aquilegifolia* (85.1%), *S. betulifolia* (84.9%), and *S. media* (84.7%) were slightly less active. The lowest reactivity towards DPPH free radicals was shown by *S. flexuosa* and *S. ussuriensis* (31.8% and 34.1%, respectively).

The Trolox equivalent antioxidant capacity (TEAC) assay revealed the extract from the first-year shoots of *S. ussuriensis* as the least active against the cation radical (13.9 µmol/L eq. Trolox/µg of the extract), while the *S. humilis* extract showed the most pronounced activity, with statistically significant differences from the other species (*p* < 0.01) of 26.7 µmol/L eq. Trolox/µg. The highest activity against the superoxide radical was shown by the *S. humilis*, *S. betulifolia*, and *S. ussuriensis* extracts, at 21.2, 21.3, and 21.7 nmol O_2_^−^/min, respectively. The extract of *S. hypericifolia* showed the least activity (29.9 nmol O_2_^−^/min), with an activity level close to that of the blank sample.

### 2.3. Antiviral Effects of the Methanolic Shoot Extracts of Spiraea spp.

The methanolic extracts obtained from the first-year shoots of 15 *Spiraea* species were screened for antiviral activity against influenza A (H1N1) virus. For this, the extracts were lyophilized and reconstituted in DMSO (concentration of the stock solutions was 50 mg/mL) and then diluted in the culture medium to obtain the final concentrations of 3.7–300 µg/mL. The cytotoxicity of the extracts was investigated and the most selective dry extracts were found (Table 3).

All of the analyzed *Spiraea* extracts showed antiviral activity against the influenza A virus. Based on the IC_50_ values, the extracts of *S. ussuriensis*, *S. chamaedryfolia*, and *S. salicifolia*, with IC_50_ values of 1, 2, and 2 µg/mL, respectively, appeared to have the highest antiviral activities. In contrast, the *S. trilobata* and *S. media* extracts yielded the highest IC_50_ values (>33 µg/mL), i.e., these extract were the least active.

Of course, the applicability of individual extracts as potential antiviral agents can be considered only in the context of the cytotoxicity data. Thus, when the cytotoxic concentration leading to the death of half of the cells in culture (CC_50_) was addressed, it was found that the most efficient extract of *S. ussuriensis* was also the most cytotoxic (CC_50_ = 19 µg/mL). On the other hand, some extracts that showed the least activity were found to be the least cytotoxic. For example, the extract from the first-year shoots of *S. trilobata* showed the least cytotoxicity (CC_50_ = 68.6 µg/mL). Therefore, the concept of selectivity (selectivity index SI = CC_50_/IC_50_) was introduced to evaluate the safety of using extracts as antiviral agents, i.e., to compare the efficacy of extracts and assess the risk of cytotoxic properties. In terms of selectivity, *Spiraea* specimens were arranged as follows: *S. media* < *S. flexuosa* = *S. hypericifolia* = *S. trilobata* < *S. crenata* = *S. elegans* = *S. sericea* < *S. pubescens* = *S. betulifolia* < *S. aquilegifolia* < *S. salicifolia f. alpestris* < *S. chamaedryfolia* < *S. salicifolia* < *S. humilis* < *S. ussuriensis*. Thus, *S. ussuriensis* (SI = 19) and *S. salicifolia* and *S. humilis* (SI = 16) showed the highest selectivity against influenza A virus. Due to this, these spirea species can be considered as the most promising candidates for potential sources of antiviral agents.

### 2.4. Antibacterial Activity of Methanolic Shoot Extracts of Spiraea spp.

The antibacterial activities of the extracts prepared from first-year shoots of the 15 *Spiraea* species were investigated in vitro by broth microdilution assay, and the corresponding MICs were determined for each of the six test microorganisms (Table 4). The assay revealed either a high (MIC < 1 mg/mL) or moderate (1 mg/mL ≤ MIC ≤ 4 mg/mL) antibacterial activity against the Gram-positive and Gram-negative bacteria strains. The extracts obtained from *S. humilis* showed the strongest antibacterial activity; the corresponding MICs obtained with all of the tested microorganisms differed from the MICs acquired for the other extracts by 1–2 orders of magnitude. The extract from *S. media* also demonstrated high antibacterial activity with MICs 2–4 times higher in comparison to those of *S. humilis*. Interestingly, the spectra of the antibacterial activity of the extracts from these two *Spiraea* species turned out to be similar: the microorganisms *P. aeruginosa* and *MRSA* (Methicillin-resistant *Staphylococcus aureus*) were the most resistant to the action of these extracts, and both extracts most efficiently inhibited the growth of *M. luteus*, while the MICs against this microorganism were the highest for most of the other extracts. The extracts from *S. aquilegifolia*, *S. betulifolia*, *S. salicifolia S. pubescens*, *S. crenata*, and *S. salicifolia f. alpestris* exhibited high activity against *E. coli.* and *S. aureus*, with only a moderate effect observed against the other microorganisms. Finally, the last group of the extracts—the isolates from *S. chamaedryfolia*, *S. trilobata*, *S. ussuriensis*, *S. flexuosa*, *S. hypericifolia*, *S. elegans*, and *S. sericea*—showed high antibacterial activity only against *E. coli.* The antibacterial activities against the other tested microorganisms, observed with the extracts from these species, appeared to be moderate.

## 3. Discussion

Here, we present a comprehensive phytochemical screening of the major secondary metabolites in extracts prepared from the first-year shoots of 15 *Spiraea* species occurring in the Russian Federation. We are convinced that this report is a significant step forward in respect of the current state of research in the field. Indeed, to the best of our knowledge, this is the first metabolite profiling study, accomplished with a representative set of Russian *Spiraea* species as part of a general systematic activity-driven phytochemical approach. Thus, here, to the best of our knowledge, for the first time, we employ in parallel a metabolite profiling survey with comprehensive screening of biological activities to address Russian spirea species as promising sources of biologically active complexes for application in medical practice.

Having in mind the pharmaceutical context of this work, we focused on the structural annotation of only major secondary metabolites with further interpretation of their prospective roles as bioactive natural products based on the activity data. The selection of the major metabolites relied on 4.5 × 10^7^ counts in corresponding mass spectra as the threshold value of their signal intensity. On the other hand, we did not go into much detail regarding the comprehensive characterization of the interspecies metabolic differences at the level of minor compounds, although we realize that multiple variations in their relative contents might be found, due to the high chemical diversity of the selected set of *Spiraea* species, which is clearly seen from the relative abundances of the top constituents (Figure 2). When making this decision, we relied on the general assumption that the major components of the extracts should contribute the most to their biological activity profiles. On the other hand, excluding the low-abundance species might essentially simplify the overall picture and facilitate an understanding of the structure–activity relationships (SARs) in the follow-up steps of this study.

Expectedly, the results obtained here did not conflict with the previously published data, which indicated polyphenols as the principal group of semi-polar secondary metabolites constituting methanolic extracts of *Spiraea* shoots [31,32,33,36,37]. Thus, when applying the selected criteria of secondary metabolites extracted from the first-year shoots of the fifteen *Spiraea* species considered, we annotated, in total, 33 major components. Among them, 15 metabolites represented the class of flavonoids, namely, quercetin derivatives (**2**, **4–6**, **8–9**, and **12–18**) and kaempferol derivatives (**10–11**). The presence of quercetin and kaempferol derivatives has been described previously, and both flavonoid aglycones and their derivatives (mainly *O*-glycosides) were characterized in the spirea parts as one of the major compounds [28,38,39,40,41]. On the other hand, no previously undescribed compounds were found in the studied *Spiraea* species. In addition, a wide variety of apigenin, luteolin, and isorhamnetin derivatives were reported in the leaves and shoots of various spirea species [36,42,43]. However, these metabolites, as well as some isoflavones, reported previously in *Spiraea* plants [31,44], were not discovered as the major extract components here.

The additional group of annotated major extract constituents included 15 secondary metabolites that could be attributed to hydroxycinnamic acids. This group of compounds appeared to be no less diverse and abundant (at least based on the intensity of the corresponding chromatographic signals) than the flavonoids. Among the major metabolites of the studied species, derivatives of caffeic (1, 7, 22, and 28), coumaric (19, 24, 26, 29, and 31) and ferulic (27 and 32) acids were tentatively annotated. A wide variety of derivatives of this group were previously reported [45,46,47]. Also, among the major components, we found derivatives of glutaric acid and geraniol alcohol, which, to the best of our knowledge, were not reported previously. In addition, three components that could not be annotated using the chromatography–mass spectrometric method were found during the study of the composition of the major components of the extracts. Apparently, additional studies are needed to identify these compounds, which include obtaining them individually and establishing their structure by nuclear magnetic resonance spectroscopy.

When comparing the profiles of the major extract components, it was found that only a minor part of them were common for all, or almost all, of the species (Figure 2). Thus, only caffeoyl-quinic acid (**1**) appeared to be the common component for all of the extracts, while quercetin hexoside (**9**) occurred in seven and coumaroyl-loganic acid (**24**) in eight out of the fifteen extracts. The other 30 identified major constituents were distributed among all of the studied extracts with minimal overlap in component composition and relative abundance (Figure 1 and Figure 2).

High-resolution mass spectrometry (HR-MS) as a profiling method used in this study allowed us to perform only a preliminary qualitative and semi-quantitative assessment of the composition of the major metabolites in the studied extracts. For the comprehensive characterization of major components, it is critically important to identify them unambiguously. This can be achieved using two strategies: (*i*) the isolation of pure compounds from the extracts and unambiguous structure assignment by NMR (nuclear magnetic resonance) spectroscopy, or (*ii*) by co-elution with authentic standards. In the next step, the isolated compounds (or commercially available authentic compounds) need to be used as the standards for absolute quantification by the standard addition approach [30]. However, the process of isolation and working up individual compounds is a very labor- and time-consuming and rather expensive procedure. Because of this, it can be justified only when the first data on the principal extract composition and profiles of biological activity are acquired and the species of interest are selected based on some correlations elucidated between the chemical composition and biological activity.

Therefore, here, we complete this first and absolutely mandatory step, and implement this logic to the study of a highly diverse taxonomic group: the genus *Spiraea*. Further in-depth investigation of the *Spiraea* species will rely on the results of this study. The rationales for selecting the species for the comprehensive studies might include the following: (*i*) the annotation of new natural products as the major extract constituents; (*ii*) the presence of significant biological activities of the extracts; (*iii*) a high natural occurrence to access sufficient resource base; or (*iv*) the availability of technologies for introduction into culture (at the cell, tissue, or agricultural levels). The latter requirement could be addressed as early as the step of selecting the species for the study. Taking into account these facts, the preliminary selection of the 15 confirmed species was made taking into account the high prevalence of these species on the territory of the Russian Federation and the possibility of their introduction into culture. To identify the most promising species for further research, the biological activity of their methanol extracts was screened using several relevant in vitro models.

Since the main group of biologically active plant compounds are representatives of the group of polyphenols, the choice of activities was addressed in favor of models, the mechanism of which is presumably related to the antioxidant properties of the studied components. This key property of the phenolics in general and polyphenols in particular allows for the targeting of oxidative stress, which is known to be associated with multiple pathologies including age-related diseases, like type 2 diabetes mellitus (T2DM) and neurodegenerative diseases [48,49,50]. Thus, the antioxidant properties of phenolics are tightly connected with an array of other biological activities, including anti-inflammatory, antidiabetic, neuroprotective, and anti-aging effects [51,52,53,54].

Taking this fact into account, the analysis of the antioxidant activity of polyphenol-containing plant extracts has become a gold standard in activity-driven phytochemical research. Therefore, much data on the antioxidant properties of plant extracts are available [55,56,57], which allows for the cross-verification of newly acquired information. Finally, due to their relative simplicity and reliability, antioxidant tests can be easily performed in a high-throughput format, being a convenient methodology for screening large amounts of biological material. In this study, we used three antioxidant activity assays (DPPH assay, TEAC assay, and NBT assay) to evaluate the activity profile of the extracts in the most comprehensive way. The choice of these three models was based on their complementarity and the possibility of using different mechanisms to understand their effects on different pathways of ROS metabolism. The data obtained showed that the most pronounced antioxidant properties in all three experiments (99.5% in the DPPH test, 26.7 µmol/L eq. Trolox/µg in the TEAC test, and 21.2 nmol O_2_^−^/min in the NBT test) were shown by the extract of *S. humilis*, which contained several derivatives of quercetin, caffeic acid, coumaric acid, and geraniol as the major constituents. Based on their activity, the rest of the analyzed extracts could be divided into two groups: the extracts with high activity and the extracts with moderate antioxidant activity. The methanol extracts of *S. aquilegifolia*, *S. trilobata*, *S. betulifolia*, *S. media*, and *S. salicifolia* can be classified into the first group. The extracts of other species of the genus—*S. ussuriensis*, *S. chamaedryfolia*, *S. pubescens*, *S. crenata*, *S. flexuosa*, *S. hypericifolia*, *S. elegans*, *S. sericea*, and *S. salicifolia f. alpestris*—were classified as moderately active.

In general, these results are consistent with the published data. The high antioxidant activity of some species of the genus has been noted earlier [21,58]. A relatively high activity of methanol extracts of *S. canescens* against free radical and superoxide anion radical comparable to the activity of the used standard n-propyl gallate has previously been shown. The activity of Far Eastern species of the *Spiraea* genus such as *S. betulifolia*, *S. betulifolia*, *S. humilis*, *S. salicifolia*, *S. pubescens*, and *S. media* was also previously shown by rapid amperometry. In addition, the antioxidant effect of *S. prunifolia* var. *simpliciflora* was shown to be comparable to its superoxide dismutase activity [28]. It was noted that, in general, previously published indicators of antioxidant activity of various species of the genus *Spiraea* do not contradict the data obtained in the course of this work and indicate a relatively high antioxidant potential of representatives of the genus. At the same time, no general patterns in the qualitative and relative quantitative composition of the major components in the studied extracts were revealed, which suggests that their unique complexes, characteristic of a particular plant species, rather than individual major compounds present in the extracts, are responsible for their activity (Figure 2). The data obtained by us agree with earlier studies describing high antioxidant potential, positively correlating with the content of polyphenolic compounds (total polyphenols, flavonoids, and phenolic acids), and also link the manifestation of the antioxidant activity of plants of this genus with the accumulation of a complex of compounds of unique compositions with a different number of hydroxyl groups directly involved in radical quenching [58,59,60]. The correlation analysis of the dependence of antioxidant activity on the content of metabolites carried out in our analysis showed a significant contribution of phenolic acid derivatives (geranyl pentosyl hexoside, caffeoyl-loganic acid, and hexopyranosyl-hexanoyl-hexapyranose) and some quercetin derivatives (quercetin acetyl-hexosyl-deoxy-hexoside) to the manifestation of different types of antioxidant activity. Thus, geranyl-pentosyl-hexoside, caffeoyl-loganic acid, quercetin acetyl-hexosyl-deoxy-hexoside, and hexopyranosyl-hexanoyl-hexapyranose made the greatest contribution to the display of activity against cation radicals in the TEAC test (the Pearson correlation coefficients were 0.69, 0.68, 0.66, 0.64, and 0.64, respectively); geranyl-pentosyl-hexoside and hexopyranosyl-hexanoyl-hexapyranose contributed the most to the free radical activity in the DPPH test (the Pearson correlation coefficients were 0.63 and 0.62, respectively); and quercetin malonyl hexoside contributed the most to the superoxide anion radical activity in the NBT test (the Pearson correlation coefficient was 0.59) (Figure S3). Consequently, the high content of these components in the extracts of *S. humilis*, *S. hypericifolia*, *S. elegans*, and *S. trilobata* may account for the high antioxidant potential of these plant species.

At the next step, we established the antiviral activity profiles of the spirea extracts against influenza A virus. Influenza is one of the most common viral pathologies with high mortality and risk of systemic complications [61,62]. It should be noted that there is currently a wide range of drugs available on the pharmaceutical market for the prevention and treatment of viral diseases. However, the vast majority of the medicines presented belong to the group of symptomatic and immunomodulatory preparations, while there are unjustifiably few direct-acting antiviral agents with proven efficiency. Therefore, research aiming for the development of new efficient drugs for the individual and combination therapy of influenza remains an important public health challenge. Earlier studies demonstrate the high activity of polyphenol-rich extracts of various plant species against influenza virus types A and B [63,64,65].

Earlier studies on Asian species of the genus *Spiraea* on the inhibition of the replication of human influenza virus A/Aichi/2/68 (H3N2) and avian influenza virus A/chicken/Kurgan/05/2005 (H5N1) in MDCK cell culture showed the presence of antiviral activity of different degrees of severity. *S. hypericifolia* extract was selected as the most promising antiviral agent in this study, having the highest effect against both human and avian influenza virus. In addition, a significant effect was observed in *S. alpina* and *S. crenata* against the H5N1 strain, and in *S. pubescens*, *S. betulifolia*, *S. media*, *S. salicifolia*, and *S. dahurica* against the H3N2 strain virus [66].

Typically, such antiviral activity relies on several types of mechanisms. For example, polyphenolic compounds prevent virus attachment to the cell membrane by binding to surface receptors; block the translocation of viral ribonculeoproteins; reduce viral protein expression, as in the case of resveratrol; or inhibit replication in the case of quercetin and its derivatives [67]. In our study, all of the extracts under consideration had moderate and pronounced antiviral activity against influenza A virus, but the highest activity was characteristic of the *S. ussuriensis*, *S. salicifolia*, and *S. humilis* extracts. These extracts were characterized by the presence of a large number of compounds from the phenolic acid group among the major metabolites. Thus, the high contents of caffeic acid, coumaric acid, and their derivatives in the extracts, along with representatives of the class of polyphenolic compounds, is capable of exerting a pronounced anti-influenza effect both when used individually and in combination with other pharmaceutical agents due to the inhibition of neuraminidase [68,69,70]. In addition, based on our correlation analysis, it was shown that the antiviral activity correlated with the antioxidant activity inherent in the studied extracts, especially regarding the activity against free radicals (the Pearson correlation coefficient was 0.66) and activity against cation radicals (the Pearson correlation coefficient was 0.60). These correlations may indicate the contribution of the antioxidant potential of the studied extracts to the mechanism of their antiviral action, which does not contradict the available literature data (Figure S4) [71]. However, to establish the exact mechanism of action of the extracts and major components of spirea extracts, it is necessary to perform additional studies.

The uncontrolled use of antibiotics by consumers, their improper disposal, their widespread use in agricultural practice, and technogenic accidents at production facilities expands the problem of microbial resistance formation to a global scale [72]. Polyphenolic compounds are widely known antimicrobial agents due to their ability to both directly affect pathogenic and opportunistic microbes and inhibit microbial virulence factors [73,74,75,76]. This is why the study of antimicrobial agents of plant origin for the creation of new antimicrobial therapy agents, as well as auxiliary agents of classical antibiotic therapy, remains a steadily relevant task. In spite of the rather long-established knowledge about the prevalence of polyphenolic compounds in plants of the genus *Spiraea*, studies of their antimicrobial activity are rather scarce. Thus, to date, data on the antimicrobial activity of species of the genus *Spiraea* are limited to screening the activity of the shoots of *S. chamaedryfolia* against *B. subtilis* (ATCC 6633), *S. aureus* (ATCC 29213), *S. pneumoniae* (ATCC 49619), *M. catarrhalis* (ATCC 25238), and MRSA (ATCC 43300); the demonstration of moderate activity of the seed extract of *S. tomentosa* against *S. aureus* (ATCC 12600), *E. coli* (ATCC 8677), *P. aeruginosa* (ATCC 9721), and *C. albicans* (ATCC 10231); and the high activity of *S. thunbergii* leaf extract against *E. coli* [21,22,28,77].

The topic of antimicrobial activity in the key extracts of representatives of *Spiraea* species has also been addressed previously. In previous studies, the significant activity of *S. tomentosa* seed extracts against Gram-positive and Gram-negative strains of microorganisms has been noted. *S. albicans* extract, in one study, showed moderate activity, but it would be incorrect to compare the data obtained by the authors with the activity data of our study, due to their use of extracts from different morphological groups of raw materials [21]. The antimicrobial activity of extracts from the aboveground part of *S. chamaedryfolia* has also been previously shown, but it would be incorrect to compare the data of the published study with the results obtained in this work due to the use of different study models [78]. There are also data in the literature on the activity of *S. thunbergii* and its metabolite butyrolactone [S-(-)-tulpalin B] against *E. coli* [22].

The screening of methanol extracts of 15 species of the genus *Spiraea* in this study showed, on average, high to moderate antimicrobial activity against Gram-positive and Gram-negative bacteria. The lowest MICs, and hence the highest antimicrobial activities against all the microorganisms tested, were possessed by the extracts of *S. humilis* and *S. media*, which are enriched with quercetin derivatives. In addition, the high antimicrobial activity of *S. humilis* may be related to the presence of geraniol derivatives in the major components, which, according to literature data, has pronounced antibacterial and antifungal activity [79,80]. It is worth noting that among the extracts studied, the geraniol derivative (compound **23**–geranyl 6-*O*-pentopyranosyl-hexopyranoside) was annotated only in the methanol extract of *S. humilis*.

Further, the correlation analysis revealed that antimicrobial activity against the selected microbial strains correlated with high contents of dihydroquercetin hexoside, quercetin malonyl hexoside, and coumaroyl hexoside derivatives in the extracts. Based on these data, it is possible to explain the relatively high activity of *S. elegans* (the species characterized by the highest dihydroquercetin hexoside content) and *S. hypericifolia* (the species characterized by the highest quercetin malonyl hexoside content) against *Escherichia coli* (MIC 125 and 250 μg/mL, respectively). However, on the other hand, the *S. ussuriensis* extract, characterized by the highest amount of coumaroyl-hexoside derivatives, responsible for the antimicrobial activity against *Pseudomonas aeruginosa*, showed no significant activity against this strain (MIC 2000 μg/mL) (Figure S3).

In addition, based on our correlation analysis, it was shown that antimicrobial activity against *Pseudomonas aeruginose* and *Micrococcus luteus* strains correlates with antioxidant activity. Thus, Figure S4 shows the possible correlation of antimicrobial activity against *Pseudomonas aeruginose* with activity against cation radicals (the Pearson correlation coefficient was −0.61), and the correlation of activity against *Micrococcus luteus* strain with activity against free radicals and cation radicals (the Pearson correlation coefficients were −0.66 and −0.63, respectively). The data obtained are interesting in terms of the selective correlation of the antimicrobial activity of the extracts with antioxidant activity and require further verification. However, further studies are needed to better substantiate the mechanism of the antimicrobial activity of major extract compounds and the activity of this extract component as well as possible synergistic and antagonistic interactions of the extract components.

## 4. Materials and Methods

### 4.1. Plant Material

The plant material was obtained from the 15 *Spiraea* species that are most widely spread in the territory of the Russian Federation: representatives of section Chamaedryon (*S. aquilegifolia*, *S. chamaedryfolia*, *S. crenata*, *S. elegans*, *S. flexuosa*, *S. media*, *S. pubescens*, *S. sericea*, *S. trilobata*, and *S. ussuriensis*), section Glomerati (*S. hypericifolia*; section Calospira C. Koch: *S. betulifolia*), and section Spiraria Ser. Koch (*S. betulifolia* and sections Spiraria Ser.: *S. salicifolia*, *S. salicifolia f. alpestris*, and *S. humilis*, Table 5). The plant material was collected from 15 specimens, and localization of sampling is presented in Table 5. *Spiraeas* are perennial shrubs with inflorescences and young leaves (which are most rich in low-molecular-weight phenolic secondary metabolites) located at distal parts of the current year shoots. Therefore, non-timbered shoots of the first year with inflorescences and young leaves were collected for this study. We avoided collecting woody shoots, as their bark contains high amounts of high-molecular-weight polyphenols called condensed tannins, which have principally different biological properties and might distort the biological activity patterns characteristic for low-molecular-weight phenols. The first-year shoots were collected from the middle part of the bush in the afternoon time when it was sunny and dried in darkness in well-ventilated rooms. The dried material was crushed using a ball mill (Retch MM400, Haan, Germany) and sieved through a sieve with a hole diameter of 2 mm.

### 4.2. Materials

Unless stated otherwise, materials were obtained from the following manufacturers: Carl Roth GmbH + Co. KG (Karlsruhe, Germany): formic acid (p.a., ACS); Honeywell Riedel-de-Haёn^TM^ (Seelze, Germany): acetonitrile (LC-MS-grade), methanol (LC-MS-grade). All other chemicals were purchased from Merck KGaA (Darmstadt, Germany). Water was purified in-house with a water conditioning and purification system (Millipore Milli-Q Gradient A10 system, resistance 18 mΩ/cm, Merck Millipore, Darmstadt, Germany).

### 4.3. Preparation of Extracts

Dried and crushed material of first-year shoots (5 g) was extracted with methanol (1:20 *w*/*v*) by double ultrasonic extraction at room temperature (RT). Safety fundamentals for solvent handling were followed during the work. The extracts were evaporated to dryness on a rotary evaporator (DLAB CIENTIFIC CO., LTD., Beijing, China) under reduced pressure at a temperature not exceeding 40 °C. Afterwards, the dried extracts were re-suspended in water and lyophilized (Martin Christ Alpha 1–2 LD, Osterode am Harz, The Netherlands). The residues were dissolved in DMSO to obtain a concentration of 50 mg/mL and stored at −20 °C until used.

### 4.4. Metabolite Profiling

The profiling of secondary metabolites relied on the procedure of Leonova et al. (2020) [81] with minor changes. In detail, the 500 µL of lyophilized extracts of the first-year spiraea shoots were reconstituted in 1000 µL of methanol and analyzed by reversed-phase ultra-high-performance liquid chromatography coupled online to quadrupole time-of-flight mass spectrometry (RP-UHPLC-QqTOF-MS). For this, the samples (2 µL) were injected (partial injection mode) in a Waters ACQUITY I-Class UPLC System consisting of Binary Solvent Manager, FL Sample Manager, and separated at 400 µL/min on a Waters ACQUITY UPLC BEH C18 column (2.1 × 50 mm, particle size 1.7 µm, Waters GmbH, Eichborn, Germany) at 40 °C. The separation relied on the linear gradient elution mode. Eluents A and B were ultrapure water and acetonitrile with 0.1% (*v*/*v*) formic acid. After a one-min wash-out of the unbound fraction with 5% eluent B, the samples were separated in a linear gradient to 95% eluent B within 10 min. Afterwards, the column was washed with 95% eluent B for 2 min and re-equilibrated at 5% eluent B for 4 min. The column effluents were infused online in a hybrid QqTOF mass spectrometer (Sciex TripleTOF 6600, AB Sciex, Darmstadt, Germany) operated in negative ion mode using a sequential window acquisition of all theoretical mass spectra (SWATH) algorithm. The nebulizer (GS1), drying (GS2), and curtain (CUR) gases were set to 60, 70, and 55 psig, respectively, while the ion spray voltage was set to −4500 V. The MS experiments were performed in the TOF-scan mode (accumulation time 100 ms) in the *m*/*z* range of 65–1250. The tandem mass spectrometric (MS/MS and MS2) experiments were performed in the SWATH mode. Thereby, the overall *m*/*z* range (65–1250 *m*/*z*) was split in 15 SWATH windows (Q1 mass ranges) of 81 *m*/*z* each with an overlap of 1 *m*/*z*. Each *m*/*z* window was acquired with 60 ms accumulation time at the collision potential (CE) of −45 V with a collision energy spread (CES) of 35 V and declustering potential (DP) of −35 V. Nitrogen was used as collision-activated dissociation (CAD) gas. Annotation of the individual analytes relied on literature data and manual interpretation of the fragmentation patterns obtained in the MS/MS experiments.

### 4.5. Targeted Tandem Mass Spectrometry (MS/MS) Experiments

For all features, which were annotated based on the signals of corresponding [M−H]^−^ ions observed in the mass spectra (MS1) to polyphenolic structures with mass accuracy better than 10 ppm, but did not yield unambiguously interpretable fragmentation patterns in SWATH mode (typically due to simultaneous fragmentation of two or more intense *m*/*z*), additional targeted RP-UHPLC-MS/MS experiments were performed with a TripleTOF 6600 mass spectrometer (AB Sciex, Darmstadt, Germany) using the LC conditions summarized in Table S1 and source settings described in the previous section. The MS/MS conditions were set as follows: each analysis was performed with 50 ms accumulation time at the range of collision potential from −10 V to −82.5 V with collision energy spread (CES) of 0 V and declustering potential (DP) of −35 V. Nitrogen was employed as collision-activated dissociation (CAD) gas.

### 4.6. Antioxidant Effects

The antioxidant effects of the plant isolates (fractions of total extract and individual compounds) were measured using 2,2-diphenyl-1-picrylhydrazyl (DPPH) free radical scavenging, Trolox equivalent antioxidant capacity (TEAC), and nitroblue tetrazolium (NBT) assays. They were performed according to the method of Orlova et al. [37] with minor modifications, as follows.

#### 4.6.1. DPPH Free Radical Scavenging Effect

Freeze-dried methanolic extracts were solubilized in DMSO (dimethyl sulfoxide) at a concentration of 50 mg/mL. The aliquots of each sample were 50-fold diluted (final concentration 1 mg/mL) and 20 µL of these solutions (20 µg in total) was added to 1 mL portions of 40 μmol/L methanolic solution of stable nitrogen centered free radical DPPH^•^. The absorbance was monitored photometrically at 517 nm after 1 h incubation at RT. The capacity of the samples for scavenging DPPH^•^ radicals was estimated from the difference in the absorbance acquired in the presence and in absence of plant isolates. The corresponding values were expressed as the percentage of DPPH^•^ consumption as a function of the sample concentration. Thereby, the oxidant activities of the extracts were determined as relative values normalized to the antioxidant activity of ascorbic acid (taken as 100%).

#### 4.6.2. Trolox Equivalent Antioxidant Capacity (TEAC) Assay

The 2,2′-azinobis(3-ethylbenzothiazoline-6-sulfonic acid) diammonium salt (ABTS) was dissolved in water to obtain a 7 mmol/L solution, which was further oxidized to the corresponding radical cation (ABTS^+^) in the presence of 2.45 mmol/L potassium persulfate (K_2_S_2_O_8_) and incubation for 16 h at RT in the dark. The radical cation reagent (ABTS+∙) was diluted with ethanol to achieve an absorbance of 0.70 ± 0.02 AU at 734 nm. Aliquots of samples (20 µL or 20 µg in total) were added to 1 mL of the ABTS^+•^ solution. Absorbance was measured at 734 nm after six minutes of incubation in the dark at RT. Antioxidant capacities of the samples are reported as Trolox equivalents.

#### 4.6.3. Assessment of Extract Capacity to Scavenge Superoxide Anion Radicals (NBT Assay)

The stock solutions (1 mmol/L) of phenazine methosulfate (PM) in ethanol, nitro blue tetrazolium chloride (NBT) in water, and β-NADH (reduced nicotinamidadenine dinucleotide) in 0.05 mol/L phosphate buffer (pH 7.4) were freshly prepared daily. The reaction mixtures contained 73 μmol/L β-NADH, 15 μmol/L PM, 50 μmol/L NBT, and 20 μg of samples dissolved in 1 mL of 0.02 mol/L Tris-HCl buffer, pH 8.0. The absorbance was determined at 560 nm immediately after mixing the reagents and later on after 15 sec of reaction. The change in absorbance with time (ΔAbs/min) and the absorption coefficient of 1 μmol/L formazan solution 0.03 were used to calculate the rate of production of superoxide anion radicals.

### 4.7. Cytotoxicity Assay

The cytotoxic effects of the methanol extracts prepared from the shoots of the 15 selected *Spiraea* species were evaluated in an MTT cell viability assay on MDCK cell culture (ATCC CCL-34). The cells were cultured in the growth medium MEM containing 10% fetal bovine serum and passaged twice a week with 0.25% trypsin in DPBS solution. For cytotoxicity experiments, cells were stripped off the plastic, concentrated by centrifugation, resuspended in the growth medium, and the concentration of cell suspension was assessed using a Neubauer chamber. The suspension was diluted to 0.25 × 10^6^ cells /mL and 100 μL of cells were seeded in each well of a 96-well plate. The cells were left in a CO_2_ incubator for adhesion to the substrate for 24 h. On the next day, a series of extract dilutions (3.7–300 μg/mL) in serum-free MEM medium was prepared. Next, the growth medium was removed, and 200 µL of extract dilutions was added to each well of the plate in three parallels. Incubation of cells with dilutions of substances was carried out for 72 h. After incubation, the medium was removed and 200 µL of 0.5 mg/mL 3-(4,5-dimethylthiazolyl-2) 2,5-diphenyltetrazolium bromide (MTT) solution in phosphate-buffered saline was added to each well of the plate The plates were further incubated at 36 °C for 2 h. After the incubation, the medium was removed, the formazan crystals formed in the cells were dissolved in DMSO, and the intensity of staining was quantified colorimetrically by a Multiscan FC plate analyzer (Thermo Fisher Scientific, Whaltem, MA, USA) at wavelength 540 nm. Based on the data obtained, the 50% cytotoxic concentrations (CC_50_) were calculated.

### 4.8. Antiviral Assay

Viruses and cells. A/Puerto Rico/8/34 influenza virus (H1N1) was cultured in MDCK cells. The cells were seeded into 96-well plates at 10^4^ cells/well and a volume of 100 µL/well of complete MEM medium. Incubation was performed overnight in a CO_2_ incubator at 36 °C in a 5% CO_2_ atmosphere. Directly before the experiment, the cells were washed with MEM medium, and further manipulations were performed in serum-free medium.

Antiviral activity of the extracts. Aliquots of individual extracts (100 µL) were added to the wells of a 96-well microtiter plate, which were pre-covered with a monolayer of MDCK cells. The plates with cells were incubated under an atmosphere of 5% CO_2_ at 36 °C for 1 h. Afterwards, 0.1 mL of the virus suspension (m.o.i. 0.01 TCID50 per cell) in alpha-MEM medium was added to each well and incubations were continued for 72 h under an atmosphere of 5% CO_2_ at 36 °C. After the incubation, the cells were washed with MEM medium and cell viability was analyzed as described above. The 50% inhibitory concentration (IC_50_), i.e., the concentration of the compound that resulted in a 50% reduction in the cytodestructive effect of the virus, was calculated from the data obtained. Clinically approved anti-influenza drugs, rimantadine and oseltamivir carboxylate, were used as reference compounds.

### 4.9. Antibacterial Assay

The minimal inhibitory concentrations (MICs) of the extracts were determined by the microdilution broth method, as recommended by the Clinical Laboratory Standards Institute, USA, with minor changes [30]. The following bacteria strains were cultured under aerobic conditions according to the approved standard protocol: *Escherichia coli* ATCC 25922, *Pseudomonas aeruginosa* ATCC 27853, *Listeria monocytogenes* EGD, *Staphylococcus aureus* ATCC 25923, *MRSA* ATCC 33591, and *Micrococcus luteus* CIP A270. Strains *MRSA* ATCC 33591 and *Listeria monocytogenes* EGD were provided by Prof. R. Lehrer (University of California Los Angeles, USA); *Escherichia coli* ATCC 25922, *Pseudomonas aeruginosa* ATCC 27853, *Micrococcus luteus* CIP A270, and *Staphylococcus aureus* ATCC 25923 were provided by the Department of Molecular Microbiology, IEM. The microorganisms from an agar plate culture were incubated for 3–4 h in 2.1% (*w*/*v*) Mueller Hinton broth (MHB) (HiMedia, Maharashtra, India) at 37 °C on an orbital shaker at an agitation rate 150 rpm. After adjusting the turbidity to 0.5 McFarland (1.5 × 10^8^ CFU/mL), suspensions were diluted in sterile 2.1% (*w*/*v*) MHB till the final bacterial concentration of 1.0 × 10^6^ CFU/mL was reached.

The extracts from fifteen species of the genus *Spiraea* were serially two-fold-diluted (the initial concentration was 4000 µg/mL) with sterile 2.1% (*w*/*v*) MHB and 50 µL aliquots were added to the wells of a 96-well sterile polystyrene U-bottom plate (GreinerBio-one, Austria). Afterwards, 50 µL of bacterial suspension was added to each well. The controls of bacterial growth and viability and medium sterility were included. Since DMSO, used as the primary solvent for the lyophilized extracts, has its own antibacterial activity, it was shown that at the concentrations used (not more than 1%), it did not inhibit the growth of any of the tested microorganisms. The microtiter plates were incubated aerobically without shaking at 37 °C for 18 h. MICs were defined as the lowest extract concentrations that inhibited the visual growth of microorganisms. The experiments were performed in triplicates and the MICs were calculated as the medians based on the data from three independent experiments, each accompanied with the complete set of controls. As a quality control, the MIC of gentamicin for Staphylococcus aureus ATCC 25923 was determined, and the results were within the recommended limits.

### 4.10. Statistical Analysis

The numerical results were expressed as the mean (mean ± standard deviation). Statistical significance of inter-group differences was assessed by the Mann–Whitney test (*p* ≤ 0.05) with Bonferroni correction for multiple comparisons applied at the confidence level of *p* ≤ 0.05.

## 5. Conclusions

To conclude, in this study, we screened major secondary metabolites extracted with methanol from the first-year shoots of fifteen species representing the genus *Spiraea*. It was shown that the main group of metabolites in the representatives of this genus are polyphenolic compounds, and, in particular, derivatives of quercetin, kaempferol, and hydroxycinnamic acids (caffeic, coumaric, ferulic, etc.). As a result of the phytochemical and pharmacological screening, we have identified the most promising species of the genus *Spiraea* for further study, including *S. humilis*, which has a marked, complex antioxidant, antimicrobial, and antiviral effect; *S. media* with marked antioxidant, antimicrobial, and cytotoxic properties; *S. ussuriensis*, a strong antioxidant and cytotoxic species; and *S. trilobata,* with antioxidant and antiviral properties. Our results will be used for further in-depth phytochemical studies, as well as for an evaluation of the pharmacological activities of individual *Spiraea* metabolites and bioactive complexes based on their isolated purified forms. This will give access to a holistic view of the prospects for the further industrial development and application of natural products from various spirea species as biologically active additives and drugs.

## Figures and Tables

**Table 1:** Metabolites annotated in methanolic extracts of first-year shoots of fifteen species of plants of the *Spirea* genus by reversed-phase ultra-high-performance liquid chromatography–tandem mass spectrometry (RP-UHPLC-QqTOF-MS/MS).

#	t_R,_ min ^a^	[M-H]^−^ Observed (*m*/*z*) ^b^	[M-H]^−^ Theoretical (*m*/*z*) ^c^	Elemental Composition ^d^	∆m, ppm	Fragmentation Patterns ^e^	Annotation
1 ^f^	2.2	707.1816	707.1829	C_32_H_35_O_18_^−^	4.5	191.0618 (100), 353.0908 (60), 707.1861 (20)	Caffeoyl-quinic acid
2 ^f^	2.8	465.0983	465.1038	C_21_H_21_O_12_^−^	10.8	151.0042 (70), 285.0373 (50), 465.0983 (100)	Dihydroquercetin-hexoside
3 ^g^	2.9	439.1823	439.1762	C_18_H_31_O_12_^−^	0.5	131.0338 (15), 149.0442 (17), 165.0549 (10), 179.0554 (11), 191.0551 (8), 221.0658 (10), 251.0779 (5), 261.1331 (20), 311.1006 (5), 393.1762 (100), 439.1823 (40)	Hexopyranosyl-hexanoyl-hexopyranose
4 ^f^	2.9	595.1358	595.1305	C_26_H_27_O_16_^−^	9	300.0281 (20), 595.1358 (100)	Quercetin hexosyl-pentoside
5 ^f^	3.0	609.1474	609.1461	C_27_H_29_O_16_^−^	−2.1	300.0262 (40), 609.1474 (100)	Quercetin-hexosyl-deoxy-hexoside
6 ^g^	3.1	695.1402	695.1465	C_30_H_31_O_19_^−^	9.1	300.0271 (100), 651.1532 (70)	Quercetin acetyl-hexosyl-deoxy-hexoside
7 ^f^	3.1	879.2546	879.2564	C_40_H_47_O_22_^−^	2.1	161.0251 (70), 179.0361 (20), 341.0881 (15), 439.1238 (100), 879.2546 (20)	Caffeoyl-hydroxy-methylbutyryl-pentoside
8 ^f^	3.1	787.1808	787.1898	C_33_H_39_O_22_^−^	10.4	271.0258 (20), 300.0335 (70), 625.1462 (80), 787.1808 (100)	Quercetin-trihexoside
9 ^f^	3.2	927.1833	927.1837	C_42_H_39_O_24_^−^	0.4	300.0298 (40), 463.0942 (100), 927.1833 (50)	Quercetin-hexoside
10 ^g^	3.2	621.1452	621.1461	C_28_H_29_O_16_^−^	1.5	284.0336 (100), 285.0411 (40), 561.1245 (20), 579.1348 (30), 621.1452 (60)	Kaempferol-acetyl-pentopyranoside-hexopyranoside
11 ^f^	3.2	1127.2887	1127.2891	C_52_H_55_O_28_^−^	−0.2	285.0413 (40), 431.1023 (70), 563.1459 (100), 1127.2887 (10)	Kaempferol-pentosyl-deoxyhexoside
12 ^g^	3.3	679.1510	679.1516	C_30_H_31_O_18_^−^	0.9	300.0281 (10), 637.1401 (30), 679.1510 (100)	Quercetin acetyl-ethyl-hexuronide-pentoside isomer 1
13 ^f^	3.3	757.1708	757.1622	C_35_H_33_O_19_^−^	−10.3	161.0255 (10), 301.0363 (70), 455.1219 (15), 595.1360 (95), 757.1708 (100)	Caffeoyl pentoside-quercetin hexoside
14 ^f^	3.4	1099.1840	1099.1850	C_48_H_43_O_30_^−^	−0.5	300.0289 (40), 463.0878 (10), 505.1058 (100), 549.0901 (50), 1099.1840 (60)	Quercetin-malonyl-hexoside
15 ^g^	3.4	679.1506	679.1516	C_30_H_31_O_18_^−^	1.5	300.0277 (10), 637.1398 (30), 679.1506 (100)	Quercetin acetyl-ethyl-hexuronide-pentoside isomer 2
16 ^g^	3.4	741.1661	741.1672	C_35_H_33_O_18_^−^	1.5	161.0238 (20), 285.0401 (30), 301.0346 (10), 455.1181 (100), 579.1339 (60), 595.1287 (63), 741.1661 (70)	Quercetin hexopyranosyl-*O*-p-coumaroyl-pentopyranoside
17 ^f^	3.4	867.1605	867.1625	C_40_H_35_O_22_^−^	2.3	301.0365 (55), 433.0817 (100), 867.1605 (30)	Quercetin-pentoside
18 ^f^	3.4	771.1816	771.1778	C_36_H_35_O_19_^−^	−4.9	271.0255 (10), 300.0308 (90), 625.1417 (40), 771.1816 (100)	Quercetin-coumaroyl-dihexoside
19 ^g^	3.4	847.2691	847.2663	C_40_H_47_O_20_^−^	2.9	145.0292 (10), 163.0398 (5), 325.0930 (15), 423.1296 (100), 847.2691 (3)	Coumaroyl hexoside derivatives
20	3.5	537.1627	537.1614	C_25_H_29_O_13_^−^	2.5	125.0241 (20), 145.0332 (100), 231.0661 (15), 357.0968 (5), 433.1120 (5), 519.1497 (30), 537.1627 (30)	Unknown
21 ^g^	3.8	893.2781	893.2721	C_41_H_49_O_22_^−^	−6.7	147.0460 (20), 309.1000 (70), 423.1316 (20), 469.1369 (100), 893.2781 (3)	(Hydroxyphenyl)-oxopropenyl-glucopyranosyloxy-methylglutaric acid
22 ^f^	3.9	537.1627	537.1614	C_25_H_29_O_13_^−^	−2.4	135.0405 (95), 161.0240 (40), 179.0379 (100), 357.0975 (20), 519.1506 (20), 537.1627 (80)	Caffeoyl-loganic acid
23 ^g^	4.0	493.2212	493.2231	C_29_H_33_O_7_^−^	3.9	131.0349 (20), 161.0455 (18), 315.1804 (100), 447.2212 (20)	Geranyl pentosyl-hexoside derivatives
24 ^f^	4.2	1043.3410	1043.3400	C_50_H_59_O_24_^−^	−0.9	163.0417 (60), 341.1044 (60), 503.1562 (60), 521.1708 (100), 1043.3412 (40)	*p*-Coumaroyl-loganic acid isomer 1
25	4.2	983.3212	983.3343	C_52_H_55_O_19_^−^	13.3	227.1286 (5), 285.0400 (10), 389.1801 (5), 593.1303 (100), 983.3212 (5)	Unknown
26 ^g^	4.3	861.2806	861.2823	C_41_H_49_O_20_^−^	1.9	147.0458 (10), 407.1363 (20), 453.1416 (100), 861.2806 (3)	Coumaroyl hexoside derivatives
27 ^g^	4.3	505.1700	505.1715	C_25_H_29_O_11_^−^	2.9	163.0401 (10), 179.0715 (40), 205.0509 (20), 235.0616 (25), 265.0717 (40), 307.0822 (100), 325.0930 (40), 487.1593 (95), 505.1700 (60)	Coumaroyl hexoside derivatives
28 ^g^	4.4	491.1917	491.1923	C_25_H_31_O_10_^−^	1.2	179.0355 (10), 221.0462 (5), 251.0561 (10), 281.0675 (20), 341.0886 (3), 491.1917 (100)	Caffeoyl-hexopyranosy-dimethyl-octadienone
29 ^f^	4.4	521.1639	521.1664	C_25_H_29_O_12_^−^	4.8	163.0389 (20), 341.1003 (15), 505.1668 (70), 521.1639 (100)	*p*-Coumaroyl-loganic acid isomer 2
30	4.5	819.2909	819.2870	C_43_H_47_O_16_^−^	−4.8	147.0422 (100), 161.0578 (70), 189.0527 (80), 249.0708 (20), 409.1458 (95), 819.2909 (40)	Unknown
31 ^g^	4.7	475.1970	475.1974	C_25_H_31_O_9_^−^	0.8	163.0409 (10), 205.0516 (20), 325.0627 (40), 265.0727 (100), 325.0939 (80), 475.1970 (40)	Phenylbytil-*O*-dihydrocoumaroyl-hexoside
32 ^g^	4.9	667.2029	667.2032	C_34_H_35_O_14_^−^	3.9	163.0407 (40), 177.0568 (100), 265.0733 (15), 307.0836 (20), 485.1469 (90), 667.2058 (30)	Unknown derivatives of acetyl-di-feruloyl-glycerol
33 ^f^	5.8	599.1803	599.1770	C_30_H_31_O_13_^−^	−5.5	103.0558 (17), 147.0464 (100), 553.1734 (10), 599.1803 (30)	Trihydroxydihydrochalcone-siringoyl-hexoside

^a^ The analytes are listed in the order of their elution; ^b^ the *m*/*z* values were derived from the acquired spectra; ^c^ the *m*/*z* values were calculated based on the predicted elemental composition (EC); ^d^ the elemental compositions were predicted with mass tolerance of 10 ppm; ^e^ MS/MS fragmentation patterns are provided as *m*/*z* values of the fragment ions (their relative intensities); ^f^ the list of genus representatives in which the compound was found; ^f^ major metabolite annotation was performed using tandem mass spectrum acquired with a hybrid QqTOF mass spectrometer operated in the negative SWATH mode; ^g^ major metabolite annotation was performed using tandem mass spectrum acquired with a hybrid QqTOF mass spectrometer operated in the negative product ion mode with unit Q1 resolution.

**Table 2:** Antioxidant potential of the methanolic extracts obtained from the first-year shoots of *Spiraea* plants.

Plant Species	DPPH Normalized Activity, %	TEAC, µmol/L eq. Trolox/µg	NBT Assay, nmol O_2_^−^/min
*S. humilis*	99.470 ± 0.001	26.725 ± 0.007	21.240 ± 0.012
*S. aquilegifolia*	85.141 ± 0.002	19.678 ± 0.018	25.107 ± 0.006
*S. chamaedryfolia*	43.172 ± 0.002	14.218 ± 0.007	23.960 ± 0.016
*S. trilobata*	79.691 ± 0.001	21.127 ± 0.019	24.320 ± 0.019
*S. betulifolia*	84.957 ± 0.004	21.020 ± 0.007	21.342 ± 0.019
*S. ussuriensis*	34.062 ± 0.001	13.915 ± 0.005	21.693 ± 0.003
*S. media*	84.733 ± 0.001	21.664 ± 0.003	25.404 ± 0.011
*S. salicifolia*	73.227 ± 0.009	21.161 ± 0.009	22.724 ± 0.013
*S. pubescens*	56.639 ± 0.006	16.919 ± 0.008	22.920 ± 0.013
*S. crenata*	44.607 ± 0.001	14.793 ± 0.007	28.169 ± 0.016
*S. flexuosa*	31.811 ± 0.001	14.749 ± 0.005	24.071 ± 0.005
*S. hypericifolia*	74.069 ± 0.001	20.466 ± 0.015	29.969 ± 0.034
*S. elegans*	36.721 ± 0.001	14.329 ± 0.010	25.942 ± 0.007
*S. sericea*	56.337 ± 0.001	16.917 ± 0.005	27.204 ± 0.008
*S. salicifolia f. alpestris*	58.548 ± 0.002	18.752 ± 0.006	27.849 ± 0.011
DMSO (control)	-	-	30.169 ± 0.041

DPPH—2,2-diphenyl-1-picrylhydrazyl free radical scavenging assay; TEAC—Trolox equivalent antioxidant capacity; NBT (nitroblue tetrazolium) assay—assessment of capacity to scavenge superoxide anion radicals. In total, 20 µg of each lyophilized extracts was used.

**Table 3:** Antiviral activities of the methanolic extracts obtained from the first-year shoots of *Spiraea* plants.

Plant Species	CC_50_, µg/mL	IC_50_, µg/mL	SI
*S. humilis*	59.2	3.8	16
*S. aquilegifolia*	53.6	5.0	11
*S. chamaedryfolia*	27.4	2.0	14
*S. trilobata*	68.6	>33.0	2
*S. betulifolia*	50.9	13.8	4
*S. ussuriensis*	19.0	1.0	19
*S. media*	43.6	>33.0	1
*S. salicifolia*	31.1	2.0	16
*S. pubescens*	48.9	12.1	4
*S. crenata*	45.2	14.1	3
*S. flexuosa*	25.3	>11.0	2
*S. hypericifolia*	45.3	19.2	2
*S. elegans*	47.5	17.7	3
*S. sericea*	44.4	13.8	3
*S. salicifolia f. alpestris*	48.3	4.0	12
*Rimantadine*	324	61	5
*Oseltamivir carboxylate*	>60	0.15	>400

CC_50_—50% cytotoxic concentration, leading to the death of half of the cells in culture; IC_50_—50% effective concentration, which halves viral activity; SI—selectivity; the ratio of CC_50_ to IC_50_.

**Table 4:** Antibacterial activities of the methanolic extracts obtained from the first-year shoots of *Spiraea* plants.

Plant Species	Activity (MICs, µg/mL)					
	Microorganism Strain					
	*E.c.* ATCC 25922	*P.a.* ATCC 27853	*S.a.* ATCC 25923	*MRSA* ATCC 33591	*M.l.* CIP A270	*L.m.* EGD
*S. humilis*	62.5	500	125	500	31.2	125
*S.aquilegifolia*	125	2000	500	1000	1000	1000
*S.chamaedryfolia*	125	2000	1000	1000	2000	4000
*S.trilobata*	125	1000	1000	1000	2000	2000
*S.betulifolia*	125	1000	500	2000	1000	2000
*S.ussuriensis*	250	2000	1000	2000	4000	2000
*S.media*	125	1000	250	1000	125	500
*S.salicifolia*	125	1000	250	2000	1000	2000
*S.pubescens*	125	1000	250	1000	4000	2000
*S.crenata*	125	1000	500	1000	1000	2000
*S.flexuosa*	125	1000	1000	2000	4000	4000
*S.hypericifolia*	250	1000	1000	2000	4000	4000
*S.elegans*	125	2000	1000	1000	4000	2000
*S. sericea*	125	1000	1000	2000	4000	4000
*S.salicifolia f. alpestris*	125	1000	250	2000	1000	1000

Antimicrobial activities were expressed as minimal inhibitory concentrations, MICs. *E.c.*—*Escherichia coli*; *P.a.*—*Pseudomonas aeruginosa*; *L.m.*—*Listeria monocytogenes*; *S.a.*—*Staphylococcus aureus*; *MRSA*—*Methicillin-resistant Staphylococcus aureus*; *M.l.*—*Micrococcus luteus*.

**Table 5:** Sites of collection of the studied *Spiraea* specimens.

ID	Species	Localization of Sampling Site
I	*S. media*	Amur Oblast, Zeya c. env.
II	*S. betulifolia*	Experimental field of CSBG SB RAS
III	*S. salicifolia* f. *alpestris*	Experimental field of CSBG SB RAS
IV	*S. pubescens*	Transbaikal region, Kalga settl. env.
V	*S. humilis*	Khabarovsk Krai, Selikhino vill. env.
VI	*S. flexuosa*	Experimental field of CSBG SB RAS
VII	*S. hypericifolia*	Novosibirsk Oblast, Steklyannoye vill. env.
VIII	*S. aquilegifolia*	Rep. of Buryatia, Ivolginsk vill. env.
IX	*S. sericea*	Amur Oblast, Sergeevka settl. env.
X	*S. trilobata*	Rep. of Altai, Ust-Koksa vill. env.
XI	*S. ussuriensis*	Amur Oblast, Sergeevka settl. env.
XII	*S. chamaedryfolia*	Experimental field of CSBG SB RAS
XIII	*S. crenata*	Novosibirsk Oblast, Shibkovo vill. env.
XIV	*S. elegans*	Zabaykalsky Krai, Mogocha c. env.
XV	*S. salicifolia*	Experimental field of CSBG SB RAS

## Data Availability

The data presented in this study are available on request from the corresponding authors.

## Supplementary Materials

The following supporting information can be downloaded at https://www.mdpi.com/article/10.3390/ijms26083587/s1.
