# Supplementary material for: Biological Potential of Methanol Extracts from Plants of the Genus Spiraea Spreading in Russia
Source: Int J Mol Sci. 2025 Apr 10;26(8):3587. doi: 10.3390/ijms26083587 (PMC12026849; doi:10.3390/ijms26083587)
Supplement: Supplementary file 1 [file ijms-26-03587-s001.zip › ijms-3506423-supplementary.pdf]

Biological potential of methanol extracts from plants of the genus *Spiraea* growing in Russia

Supplementary Materials

Anastasia Orlova [1]*, Alena Soboleva [1], Elena Tsvetkova [2, 3], Svetlana Silinskaia [1], Yana L. Esaulkova [4], Tayiana N. Veklich [5], Vladimir V. Zarubaev [4], Anna A. Khakulova[6], Ilya R. Akberdin [7], Semyon K. Kolmykov [7], Vera A. Kostikova [8], Andrej Frolov [1]*

[1] Laboratory of Analytical Biochemistry and Biotechnology, K.A. Timiryazev Institute of Plant Physiology Russian Academy of Science, 35 Botanicheskaya str., Moscow, 127276, Russia;
[2] Department of Biochemistry, St. Petersburg State University, 7-9-11 University Emb., St. Petersburg 199034, Russia;
[3] Department of General Pathology and Pathological Physiology, Institute of Experimental Medicine, 197022 St. Petersburg, Russia;
[4] The Laboratory of Experimental Virology, St. Petersburg Pasteur Institute, 14 Mira Str., St. Petersburg 197101, Russia;
[5] Botany Laboratory, Amur Branch of Botanical Garden-Institute of the Far Eastern Branch Russian Academy of Science (AB BGI FEB RAS), Ignatievskoe Road, 2km, Blagoveshchensk, 675000, Russia;
[6] Chemical Analysis and Materials Research Core Facility Center, Research Park, Saint-Petersburg State University, 199034 Saint-Petersburg, Russia;
[7] Department of Computational Biology, Sirius University, 1 Olimpiyskiy avenue, Sochi, Krasnodar territory, 354340, Russia;
[8] Laboratory of Phytochemistry, Central Siberian Botanical Garden of Siberian Branch of Russian Academy of Sciences (CSBG SB RAS), 101 Zolotodolinskaya Str., Novosibirsk 630090, Russia.

* Corresponding Authors:
Dr. Anastasia Orlova, orlova@ifr.moscow; Tel.: +79118299027; https://orcid.org/0000-0002-7836-5785

Dr. Andrej Frolov: frolov@ifr.moscow; Tel.: +9046097095

Direction

Table S1. The conditions of ultrahigh performance liquid chromatographic (UHPLC) separation and the settings for electrospray ionization-quadrupole-time of flight mass spectrometry (ESI-QqTOF-MS) applied for the analysis of *Spiraea* secondary metabolites with Waters ACQUITY UPLC I-Class UPLC System (Waters GmbH, Eschborn, Germany) coupled online to a hybrid quadrupole-time of flight mass spectrometer (QqTOF-MS) AB Sciex TripleTOF 6600 (AB Sciex, Darmstadt, Germany) 4

Figure S1. Metabolites annotated in methanolic extracts of first-year shoots of fifteen species of plants of the *Spirea* genus by reversed phase ultra-high-performance liquid chromatography—mass spectrometry (RP-UHPLC-QqTOF-MS/MS) accomplished with a Waters ACQUITY I-Class UPLC System (Waters GmbH, Eschborn, Germany) coupled on-line to a Triple-TOF6600 hybrid mass spectrometer (Sciex, Darmstadt, Germany) operated in negative ion mode using a sequential window acquisition of all theoretical mass spectra (SWATH) algorithm. 7

Figure S2. Metabolites annotated in methanolic extracts of first-year shoots of fifteen species of plants of the *Spirea* genus by reversed phase ultra-high-performance liquid chromatography—tandem mass spectrometry (RP-UHPLC-QqTOF-MS/MS) accomplished with a Waters ACQUITY I-Class UPLC System (Waters GmbH, Eschborn, Germany) coupled on-line to a Triple-TOF6600 hybrid mass spectrometer (Sciex, Darmstadt, Germany) in the negative ion mode 24

Figure S3. Correlation analysis of the dependence of the investigated types of biological activity on the content of major components of methanol extracts of first-year shoots of the investigated species of the genus *Spireae*... 39

Figure S4. Graph of mutual dependence of manifestation of different types of biological activity of methanolic extracts of first-year shoots of plants of *Spireae* genus selected for the study. 40

Annotation of major metabolites in methanolic extracts of first year shoots of fifteen species of plants of the *Spirea* genus by reversed phase ultra-high-performance liquid chromatography—tandem mass spectrometry (RP-UHPLC-QqTOF-MS/MS) 41

Table S1. The conditions of ultrahigh performance liquid chromatographic (UHPLC) separation and the settings for electrospray ionization-quadrupole-time of flight mass spectrometry (ESI-QqTOF-MS) applied for the analysis of *Spiraea* secondary metabolites with Waters ACQUITY UPLC I-Class UPLC System (Waters GmbH, Eschborn, Germany) coupled online to a hybrid quadrupole-time of flight mass spectrometer (QqTOF-MS) AB Sciex TripleTOF 6600 (AB Sciex, Darmstadt, Germany)

Chromatography

**ACQUITY Sample Manager (SM)**	
Injection mode	PartialLoop
Injection volume	2 µL
Weak wash solvent	0.1% (*v/v*) formic acid in water
Weak wash volume	600 µL
Strong wash solvent	0.1% (*v/v*) formic acid in acetonitrile
Strong wash volume	200 µL
Target sample temperature	5.0 C
Needle overfill flush	Automatic
**Column conditions**	
Separation column	Acquity UPLC® BEH C18 (Waters) (50 x 2.1 mm, particle size 1.7 µm)
Target column temperature	40.0 C
**ACQUITY Binary Solvent Manager (BSM)**	
Eluent A	0.1% (*v/v*) formic acid in water
Eluent B	0.1% (*v/v*) formic acid in acetonitrile
Seal wash duration	5 min

Flow rate	0.4 mL/min
Elution program	5% eluent B isocratic - 1 min<br>gradient to 95% eluent B – 10 min<br>95% eluent B isocratic – 2 min<br>gradient to 5% eluent B – 0.5 min<br>5% eluent B isocratic – 3.5 min (re-equilibration)

Mass spectrometry

General	
Mass analyzer type	quadrupole-time of flight (QqTOF-MS)
Ion source	DuoSpray™ ion source
Experiment type	Sequential Windowed Acquisition of All Theoretical Fragment Ion Mass Spectra (SWATH)
Operation mode	negative
Cycle time (s)	1
Pause between ranges (ms)	1.049
Settling time (s)	0
Time bins to sum	4
Duration	17.001 min
**Ion source settings**	
Nebulizer gas (psig)	60
Drying gas (psig)	70
Curtain gas (psig)	55
Ion spray voltage (kV)	-4.5

Ion source temperature (°C)	450
**MS settings**	
Experiment type	TOF-MS
m/z range	65-1250
Accumulation time (ms)	50
Declustering potential (V)	-35
Collision potential (V)	-10.0
**MS/MS settings**	
Fragmentation mode	Collision-activated dissociation (CAD)
MS/MS experiment type	SWATH
SWATH window number	15
SWATH window width (*m/z*)	81
SWATH window overlap (*m/z*)	1
Rolling collision energy	off
Analyte type	Small molecules
Accumulation time (ms)	60
Declustering potential (V)	-35
Collision potential (V)	-45
Collision energy spread (V)	-35
Ion release delay (ms)	30
Ion release width (ms)	15

Figure S1. Metabolites annotated in methanolic extracts of first-year shoots of fifteen species of plants of the *Spirea* genus by reversed phase ultra-high-performance liquid chromatography—mass spectrometry (RP-UHPLC-QqTOF-MS/MS) accomplished with a Waters ACQUITY I-Class UPLC System (Waters GmbH, Eschborn, Germany) coupled on-line to a Triple-TOF6600 hybrid mass spectrometer (Sciex, Darmstadt, Germany) operated in negative ion mode using a sequential window acquisition of all theoretical mass spectra (SWATH) algorithm. The MS/MS spectra of individual metabolites are listed as Figure S1-1 – S1-16.

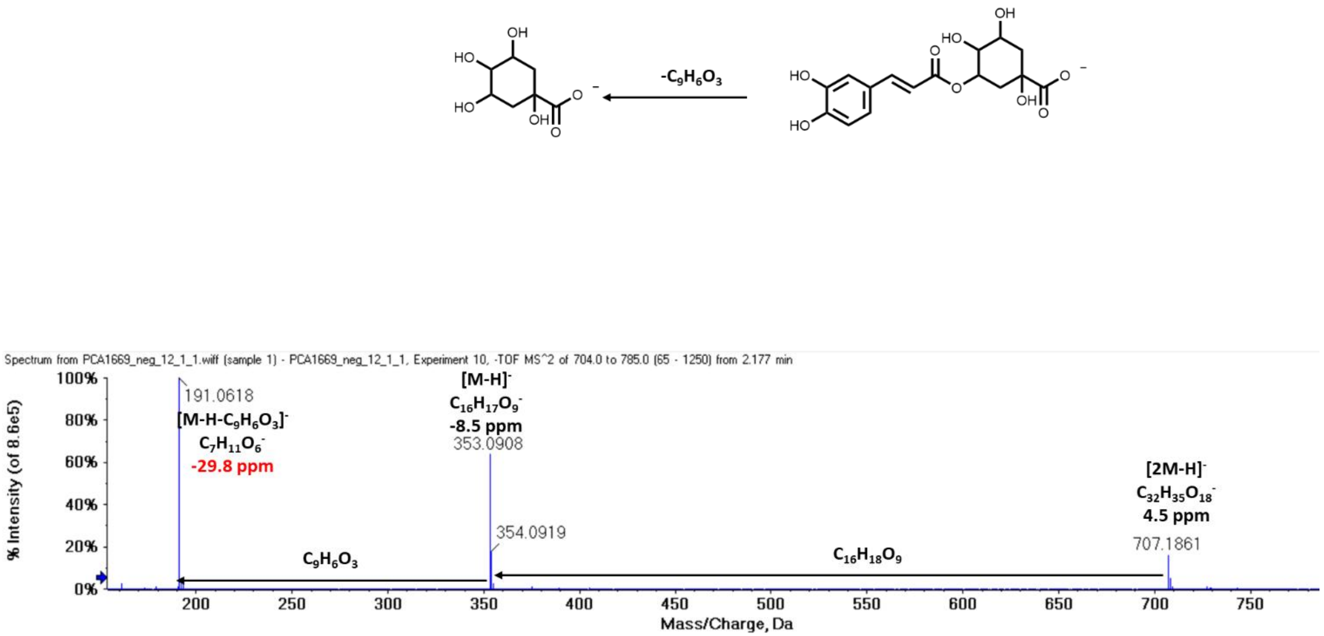

Figure S1-1. Tandem mass spectrum of *m/z* 707.2 at t_R 2.18 min corresponding to caffeoyl-quinic acid (**1**). The spectrum was acquired with a hybrid QqTOF mass spectrometer operated in the negative SWATH mode (*m/z* window 704.0-785.0). Structures shown are suggestions.

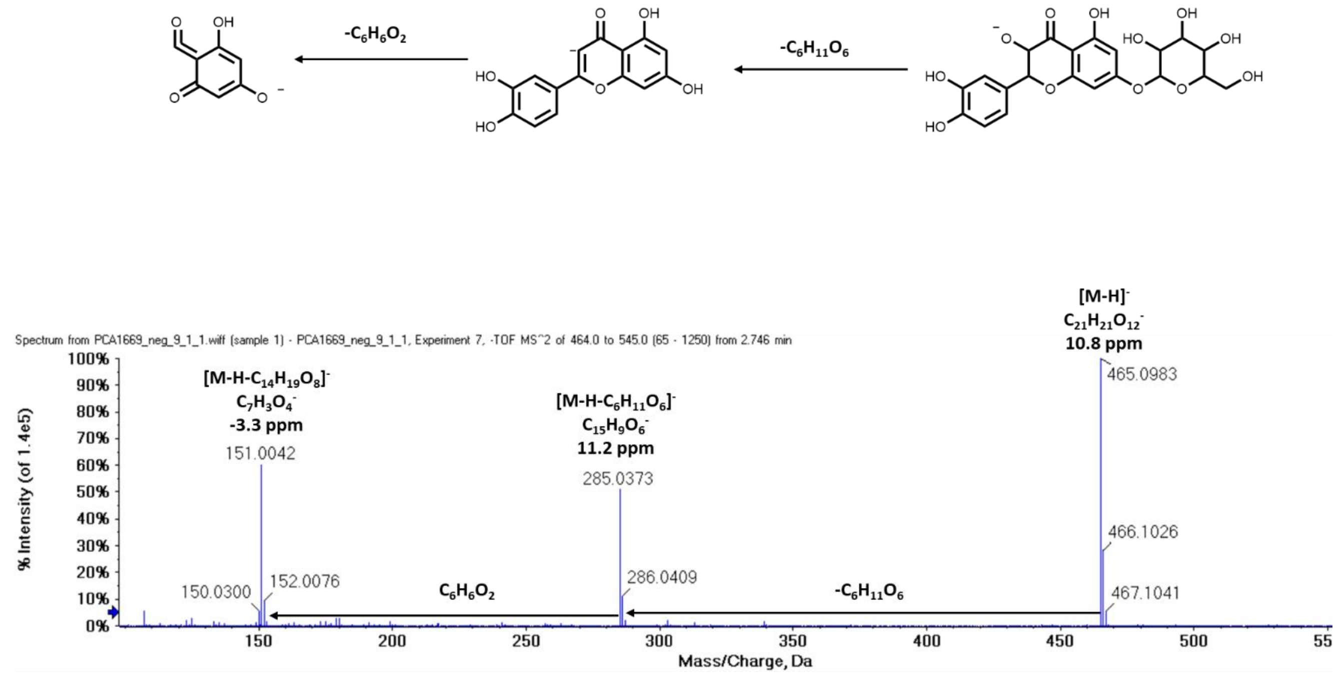

Figure S1-2. Tandem mass spectrum of *m/z* 465.1 at t_R 2.75 min corresponding to dihydroquercetin-hexoside (**2**). The spectrum was acquired with a hybrid QqTOF mass spectrometer operated in the negative SWATH mode (*m/z* window 464.0-545.0). Structures shown are suggestions.

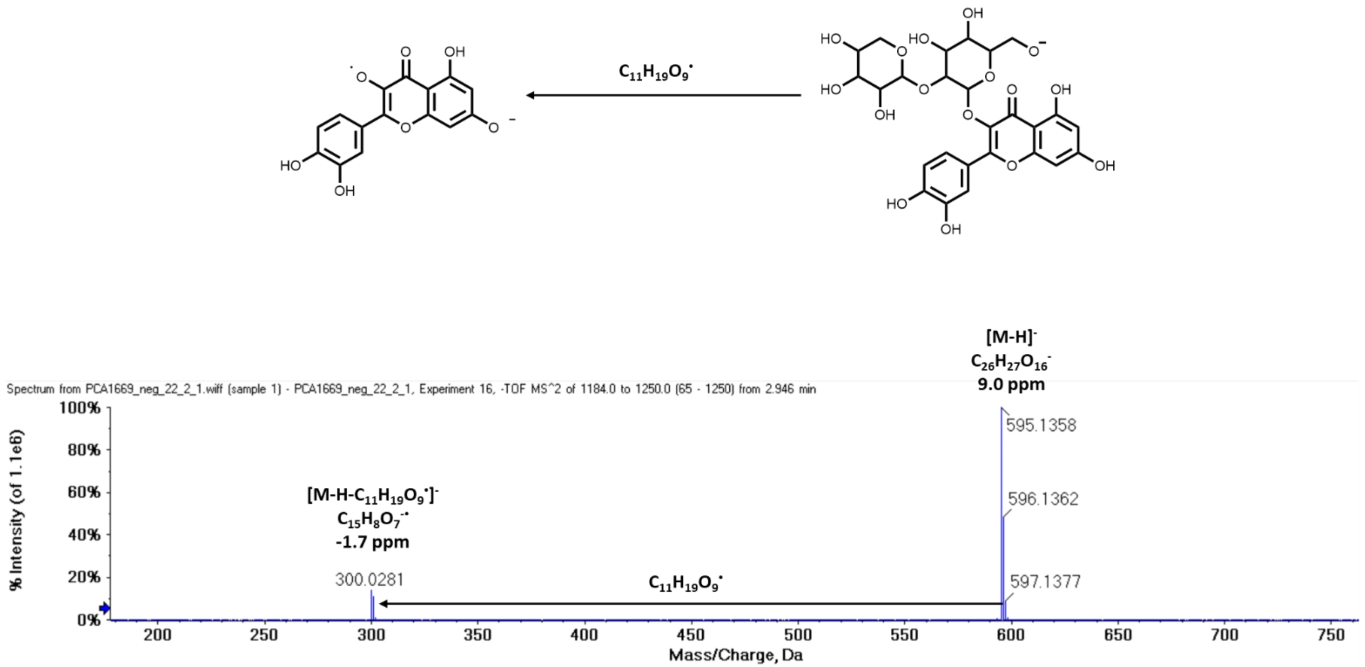

Figure S1-3. Tandem mass spectrum of *m/z* 595.1 at t_R 2.94 min corresponding to quercetin hexosyl-pentoside (**4**). The spectrum was acquired with a hybrid QqTOF mass spectrometer operated in the negative SWATH mode (*m/z* window 1184.0-1250.0). Structures shown are suggestions.

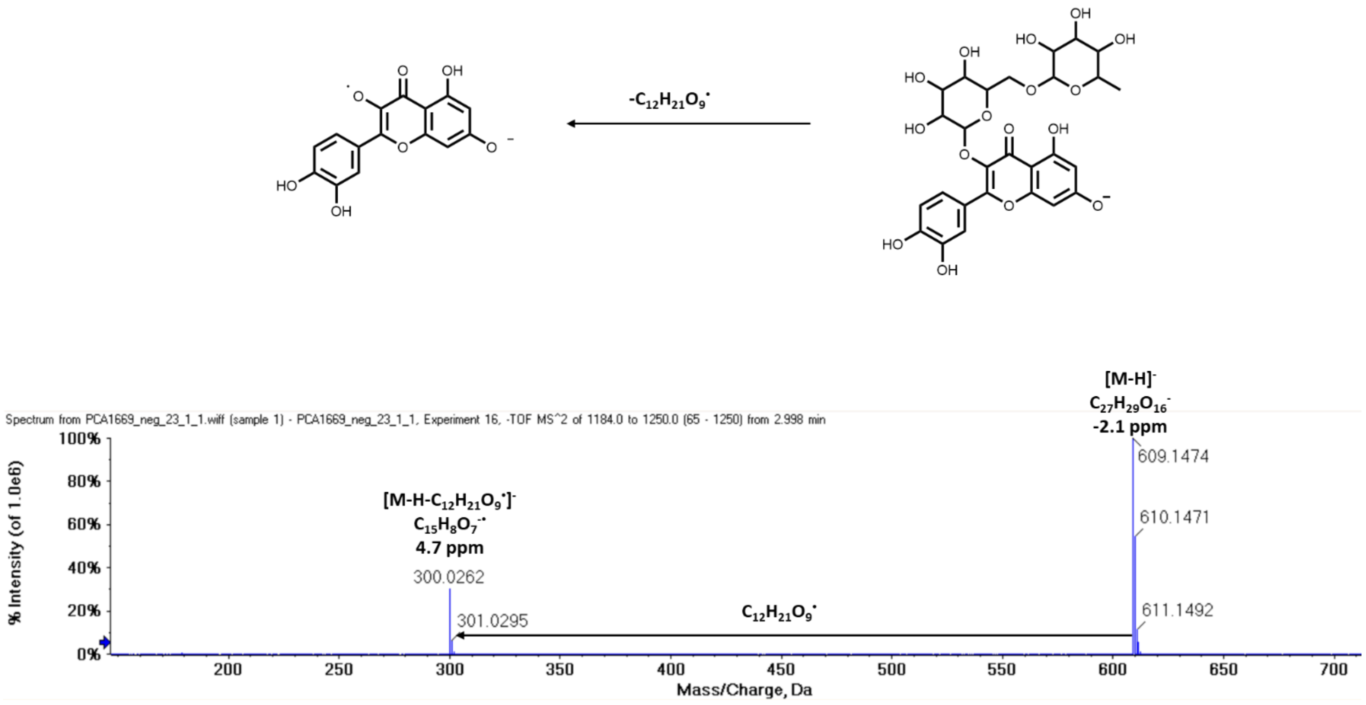

Figure S1-4. Tandem mass spectrum of *m/z* 609.1 at t_R 3.0 min corresponding to quercetin-hexosyl-deoxy-hexoside (**5**). The spectrum was acquired with a hybrid QqTOF mass spectrometer operated in the negative SWATH mode (*m/z* window 1184.0-1250.0). Structures shown are suggestions.

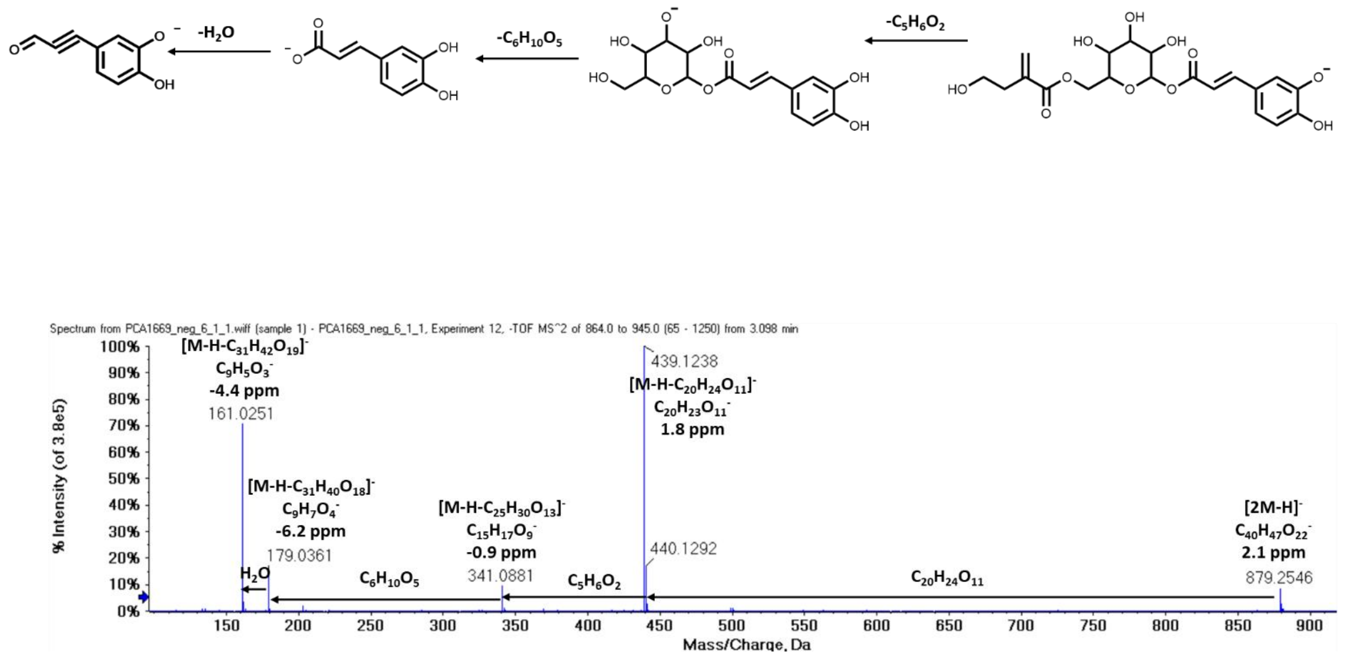

Figure S1-5. Tandem mass spectrum of *m/z* 879.3 at t_R 3.09 min corresponding to caffeoyl-hydroxy-methylbutyryl-pentoside (**7**). The spectrum was acquired with a hybrid QqTOF mass spectrometer operated in the negative SWATH mode (*m/z* window 864.0-945.0). Structures shown are suggestions.

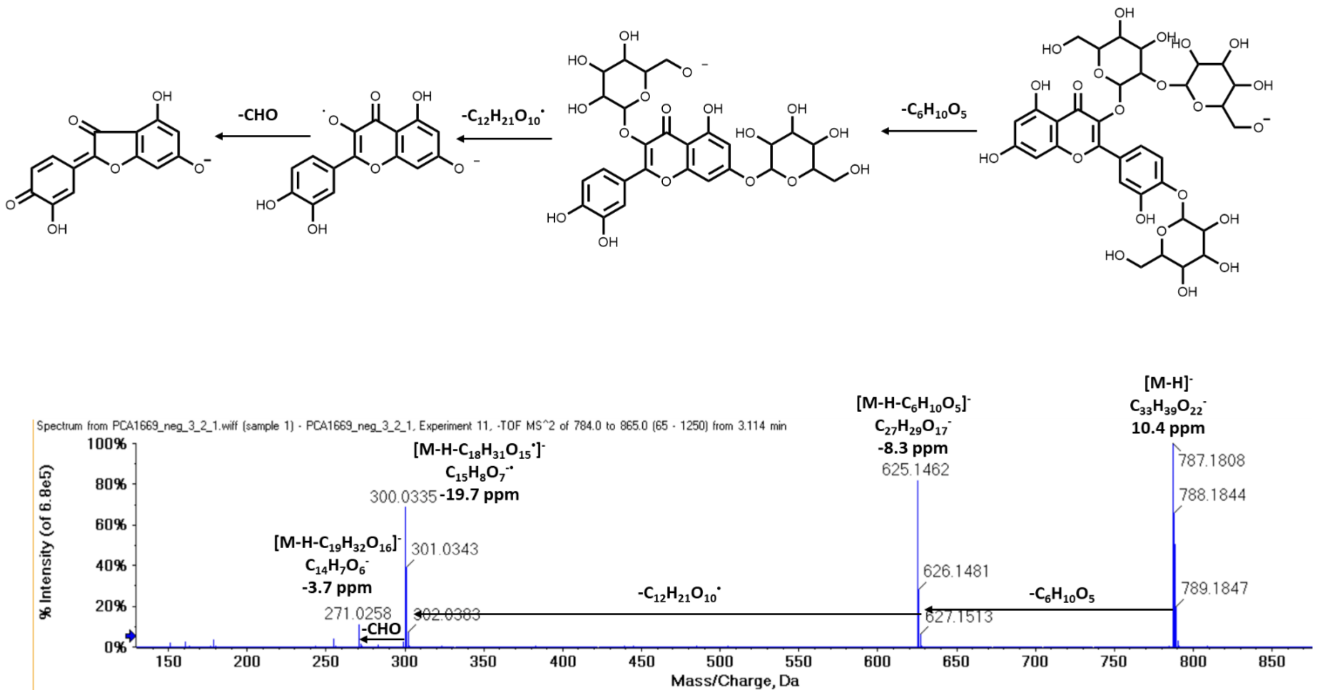

Figure S1-6. Tandem mass spectrum of *m/z* 787.2 at t_R 3.11 min corresponding to quercetin-trihexoside (**8**). The spectrum was acquired with a hybrid QqTOF mass spectrometer operated in the negative SWATH mode (*m/z* window 784.0-865.0). Structures shown are suggestions.

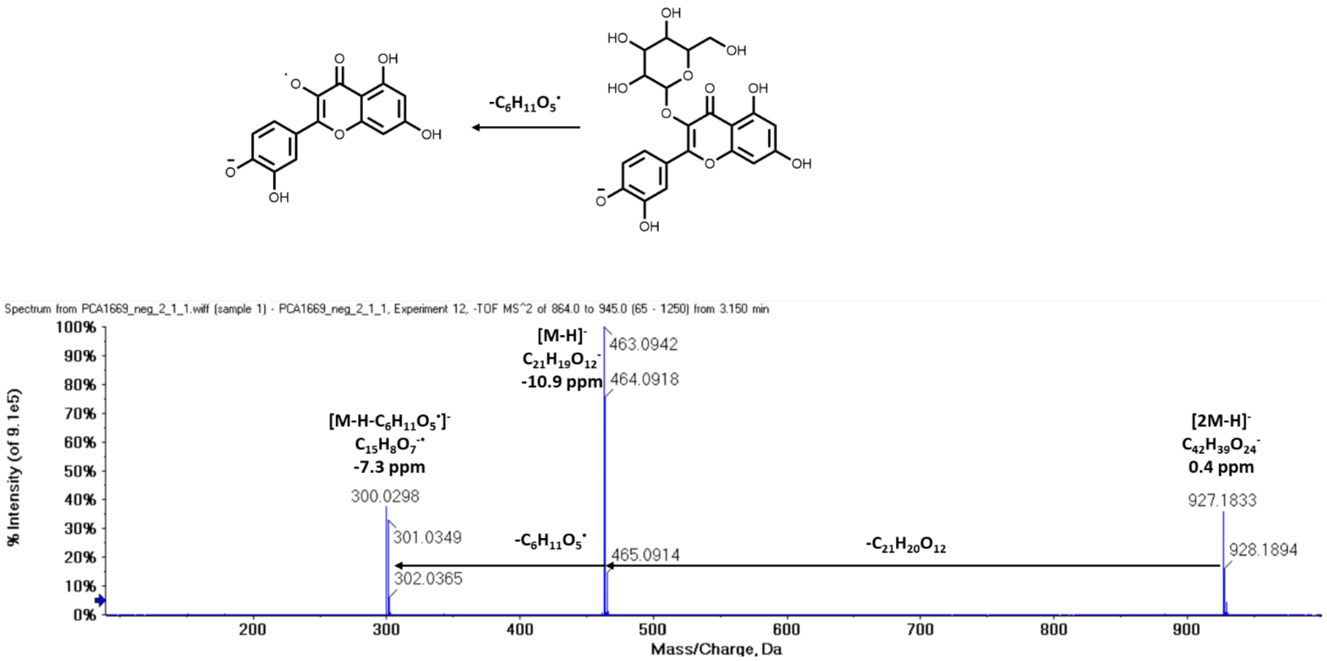

Figure S1-7. Tandem mass spectrum of *m/z* 927.2 at t_R 3.15 min corresponding to quercetin-hexoside (**9**). The spectrum was acquired with a hybrid QqTOF mass spectrometer operated in the negative SWATH mode (*m/z* window 864.0-945.0). Structures shown are suggestions.

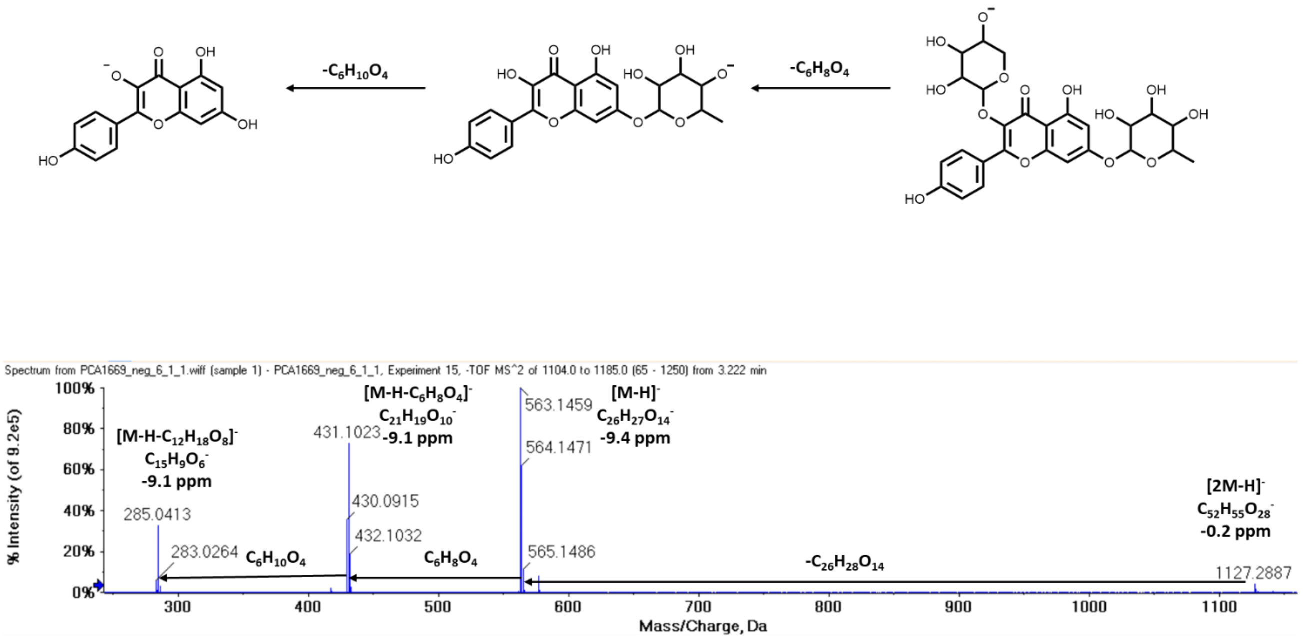

Figure S1-8. Tandem mass spectrum of *m/z* 1127.3 at t_R 3.22 min corresponding to kaempferol-dipentoside (**11**). The spectrum was acquired with a hybrid QqTOF mass spectrometer operated in the negative SWATH mode (*m/z* window 1104.0-1185.0). Structures shown are suggestions.

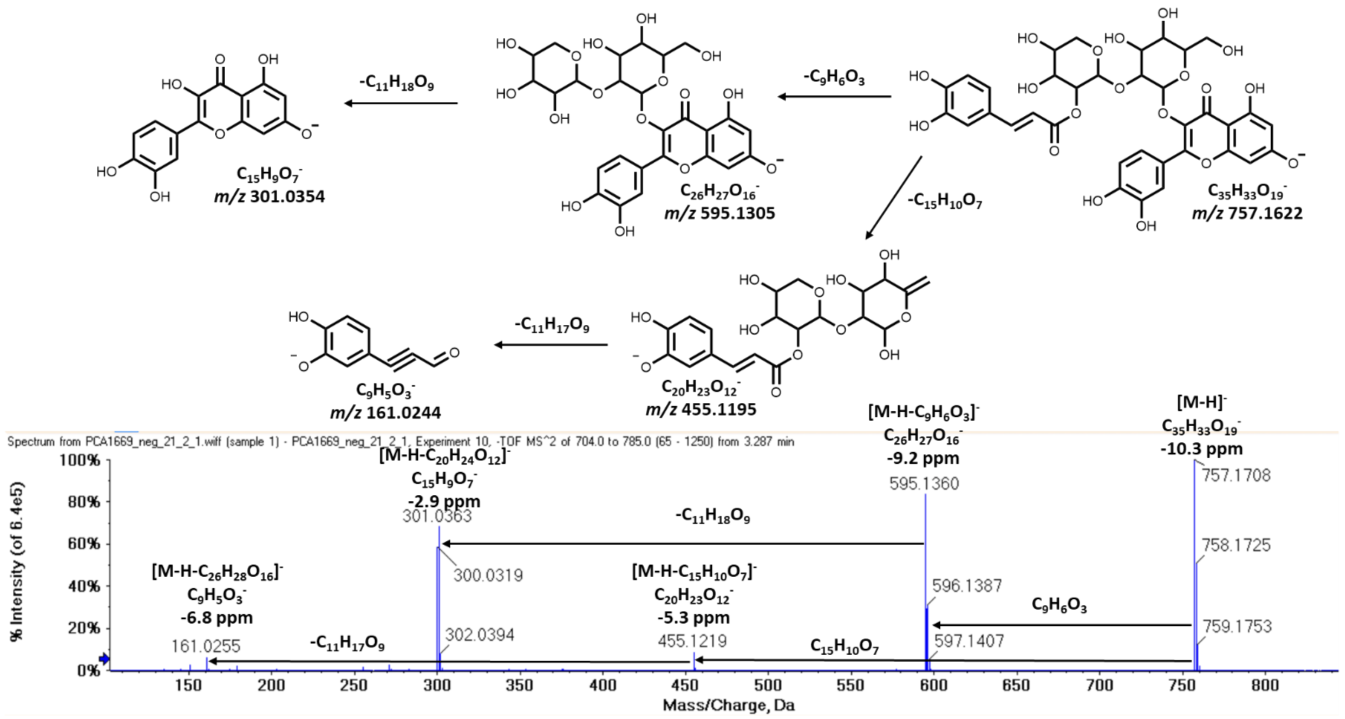

Figure S1-9. Tandem mass spectrum of *m/z* 757.2 at t_R 3.29 min corresponding to caffeoyl pentoside-quercetin hexoside (**13**). The spectrum was acquired with a hybrid QqTOF mass spectrometer operated in the negative SWATH mode (*m/z* window 704.0-785.0). Structures shown are suggestions.

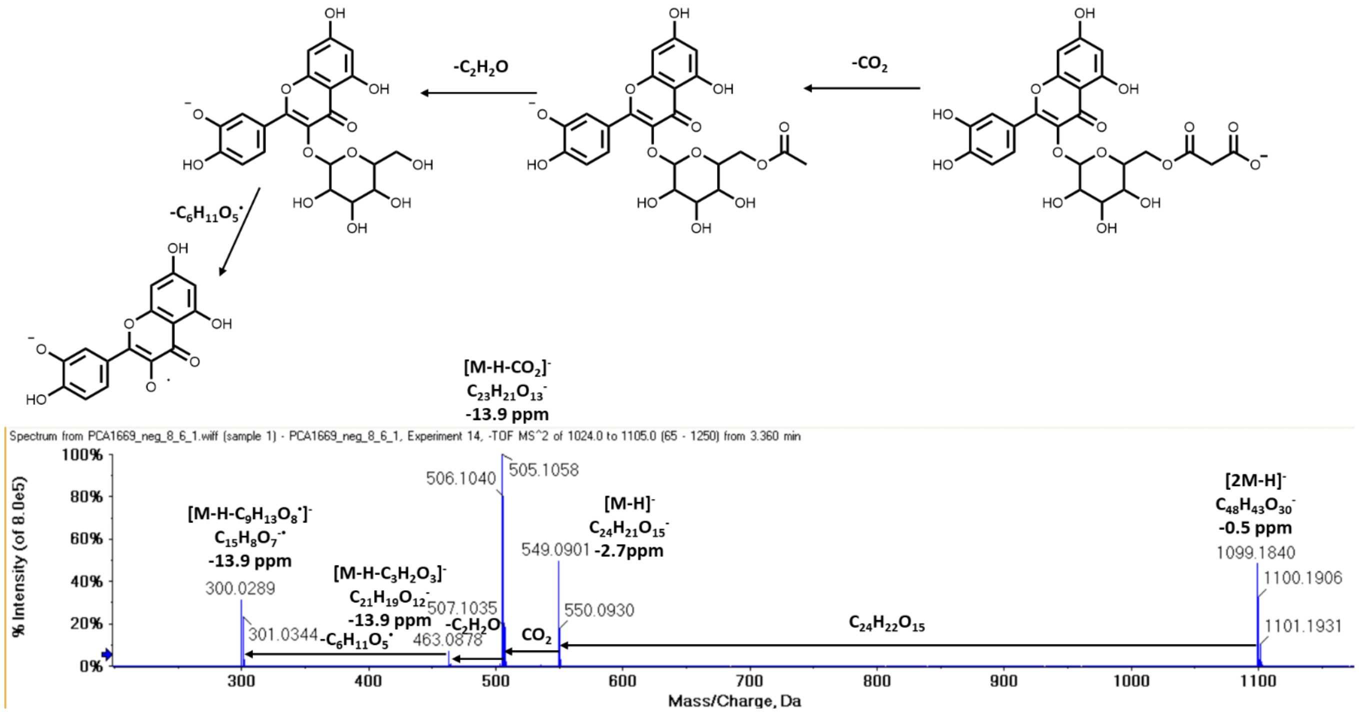

Figure S1-10. Tandem mass spectrum of *m/z* 1099.2 at t_R 3.36 min corresponding to quercetin-malonyl-hexoside (**14**). The spectrum was acquired with a hybrid QqTOF mass spectrometer operated in the negative SWATH mode (*m/z* window 1024.0-1105.0). Structures shown are suggestions.

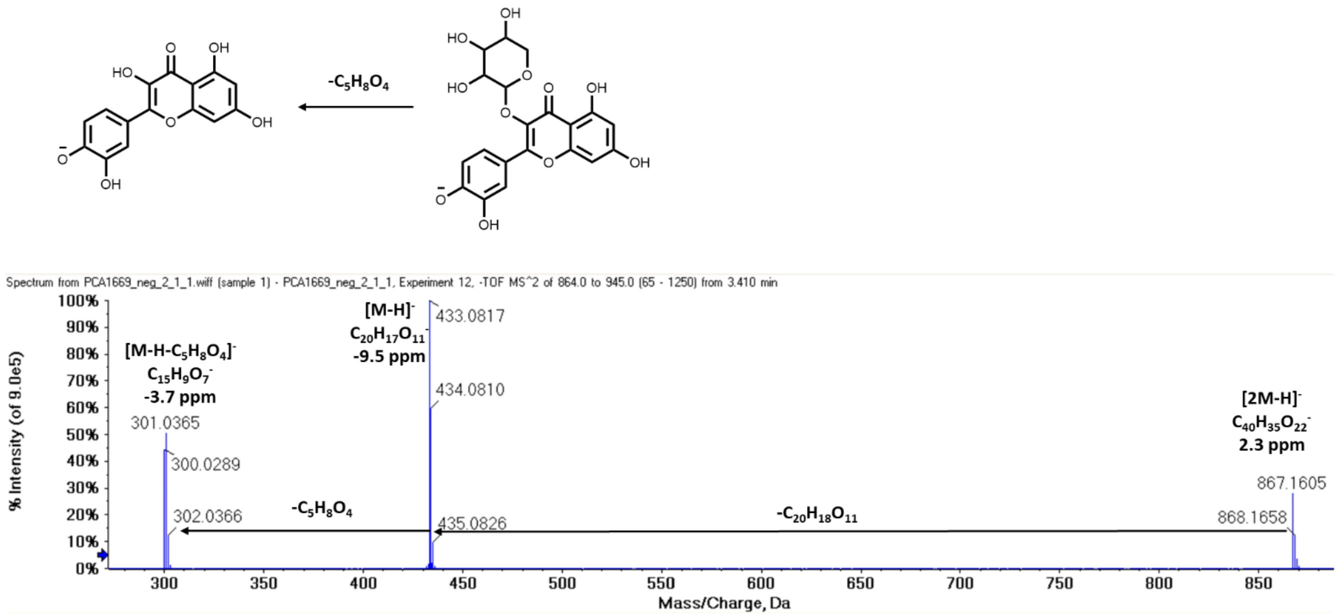

Figure S1-11. Tandem mass spectrum of *m/z* 867.2 at t_R 3.41 min corresponding to quercetin-pentoside (**17**). The spectrum was acquired with a hybrid QqTOF mass spectrometer operated in the negative SWATH mode (*m/z* window 864.0-945.0). Structures shown are suggestions.

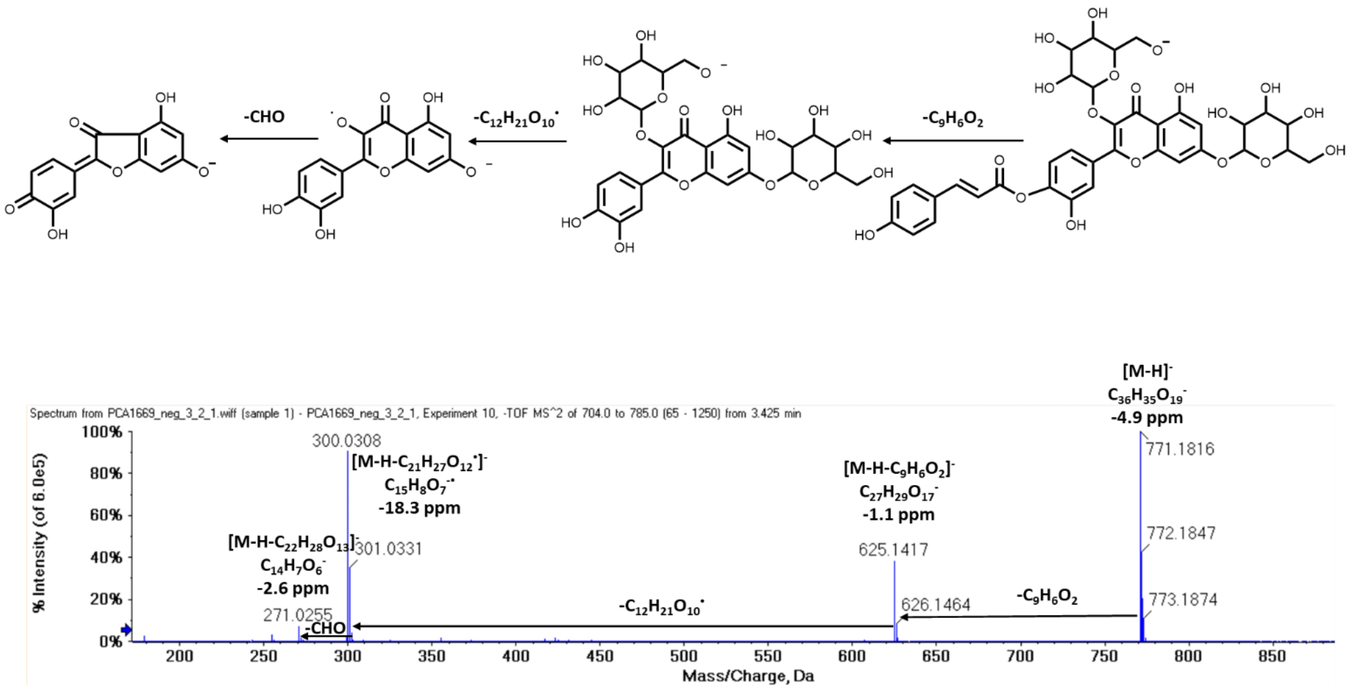

Figure S1-12. Tandem mass spectrum of *m/z* 771.2 at t_R 3.43 min corresponding to quercetin-coumaroyl-dihexoside (**18**). The spectrum was acquired with a hybrid QqTOF mass spectrometer operated in the negative SWATH mode (*m/z* window 704.0-785.0). Structures shown are suggestions.

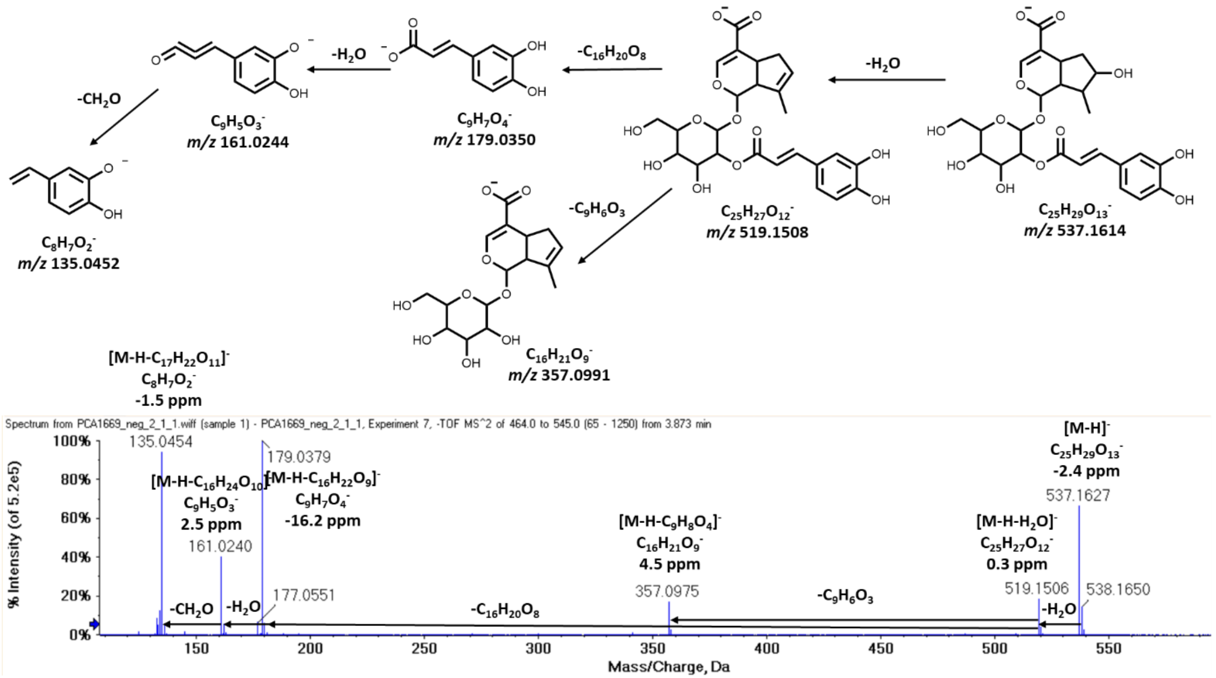

Figure S1-13. Tandem mass spectrum of *m/z* 537.2 at t_R 3.87 min corresponding to caffeoyl-loganic acid (**22**). The spectrum was acquired with a hybrid QqTOF mass spectrometer operated in the negative SWATH mode (*m/z* window 464.0-545.0). Structures shown are suggestions.

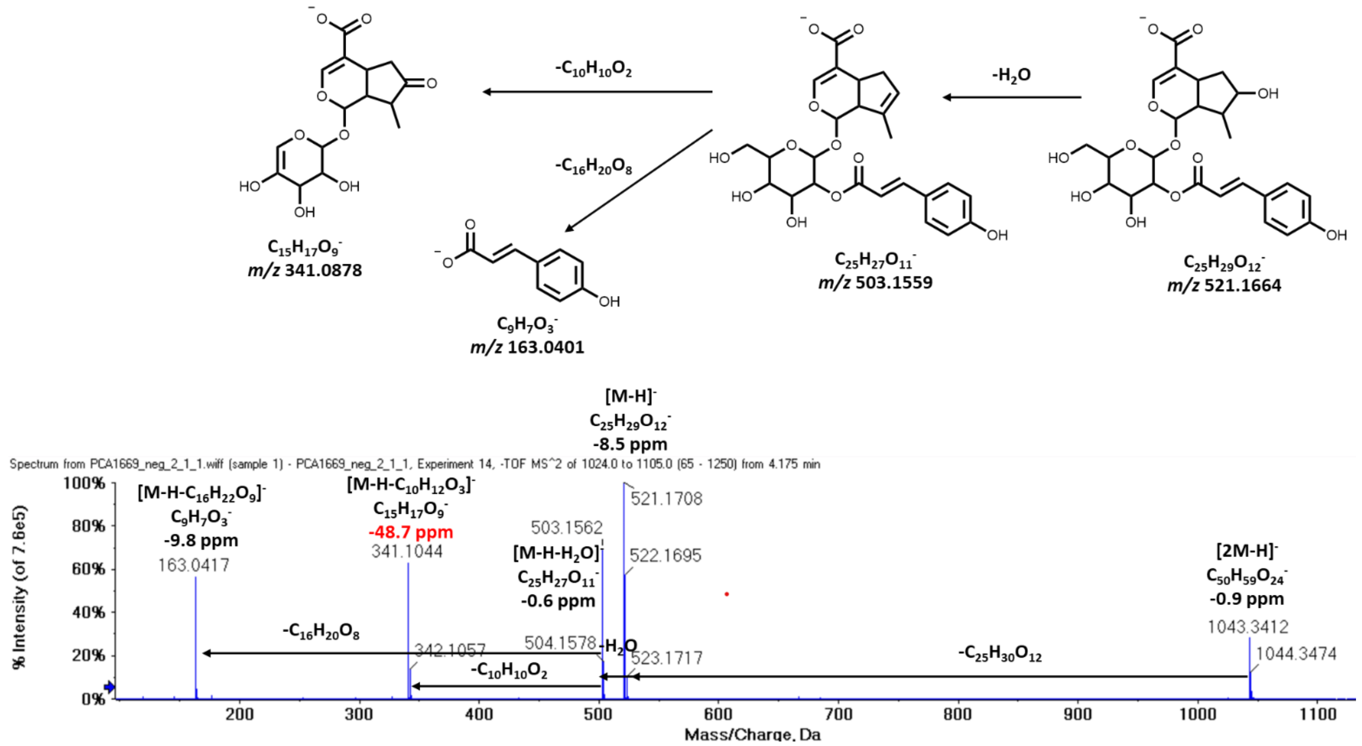

Figure S1-14. Tandem mass spectrum of *m/z* 1043.3 at t_R 4.18 min corresponding to p-coumaroyl-loganic acid isomer 1 (**24**). The spectrum was acquired with a hybrid QqTOF mass spectrometer operated in the negative SWATH mode (*m/z* window 1024.0-1105.0). Structures shown are suggestions.

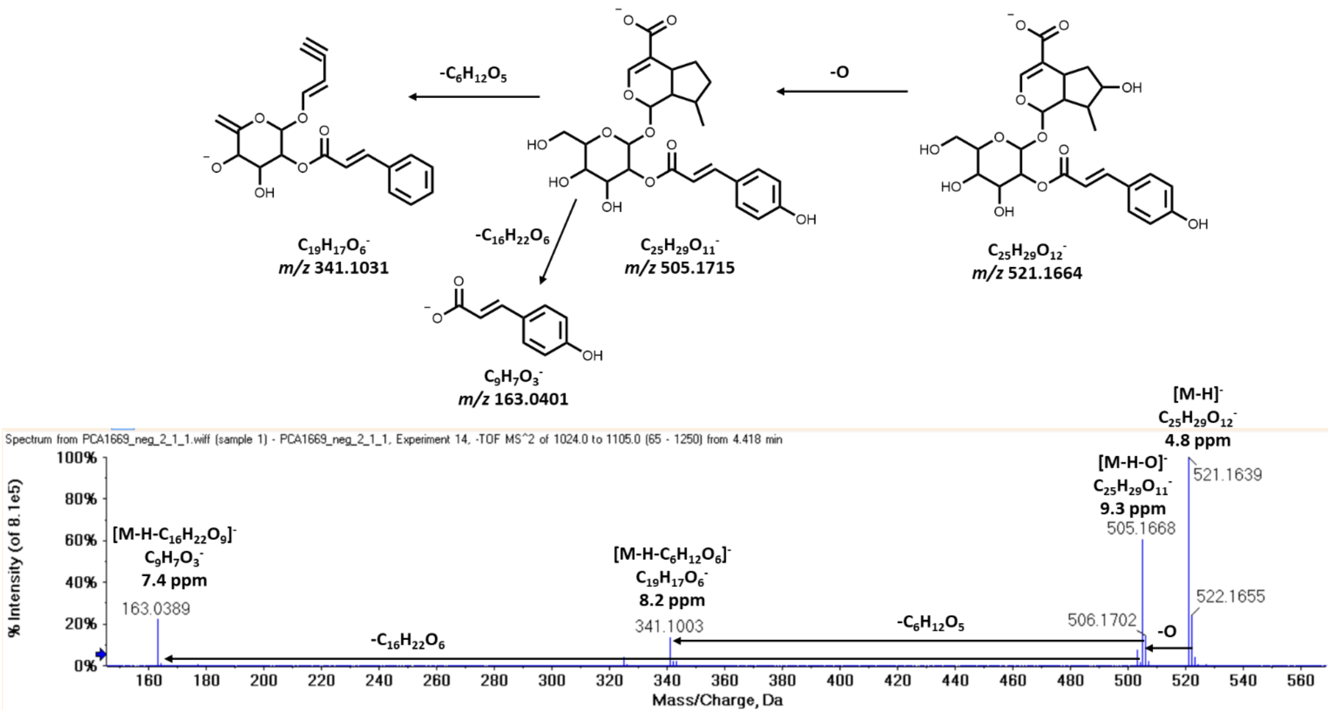

Figure S1-15. Tandem mass spectrum of *m/z* 521.2 at t_R 4.42 min corresponding to p-coumaroyl-loganic acid isomer 2 (**29**). The spectrum was acquired with a hybrid QqTOF mass spectrometer operated in the negative SWATH mode (*m/z* window 1024.0-1105.0). Structures shown are suggestions.

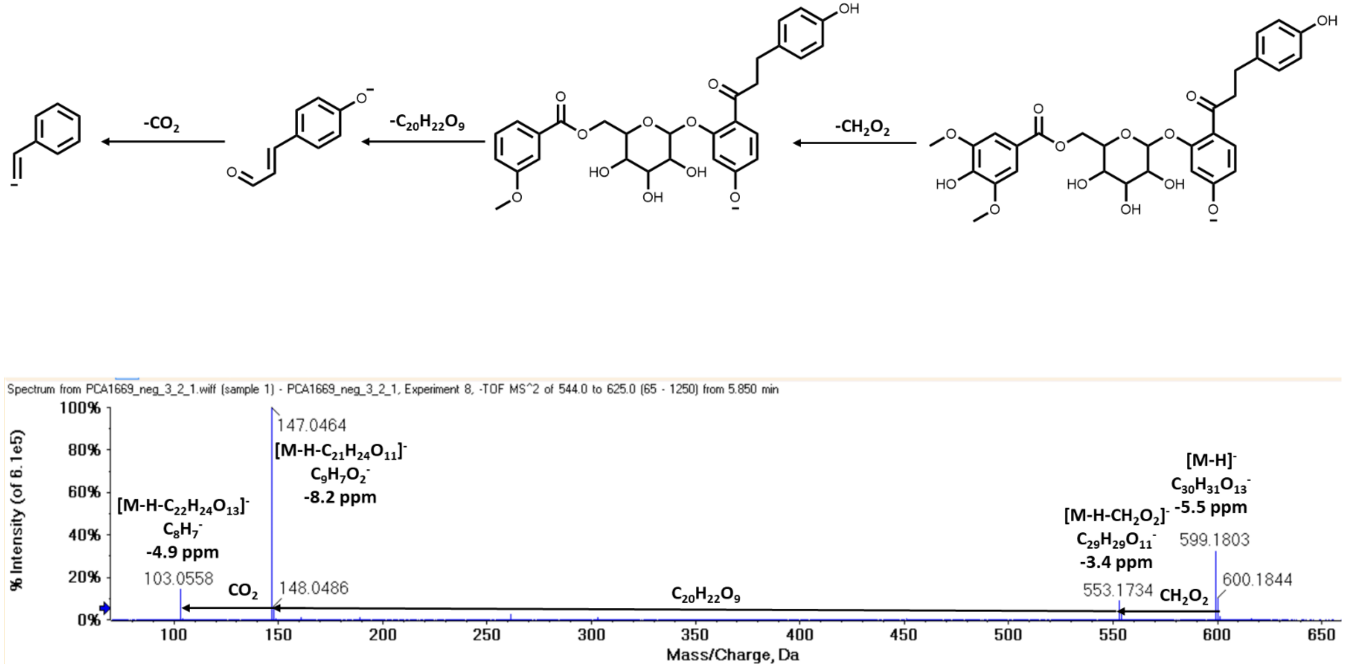

Figure S1-16. Tandem mass spectrum of *m/z* 599.2 at t_R 5.9 min corresponding to trihydroxydihydrochalcone-siringoyl-hexoside (**33**). The spectrum was acquired with a hybrid QqTOF mass spectrometer operated in the negative SWATH mode (*m/z* window 544.0-625.0). Structures shown are suggestions.

Figure S2. Metabolites annotated in methanolic extracts of first-year shoots of fifteen species of plants of the *Spirea* genus by reversed phase ultra-high-performance liquid chromatography—tandem mass spectrometry (RP-UHPLC-QqTOF-MS/MS) accomplished with a Waters ACQUITY I-Class UPLC System (Waters GmbH, Eschborn, Germany) coupled on-line to a Triple-TOF6600 hybrid mass spectrometer (Sciex, Darmstadt, Germany) in the negative ion mode. The MS/MS spectra of individual metabolites are listed as Figure S2-1 – S2-14.

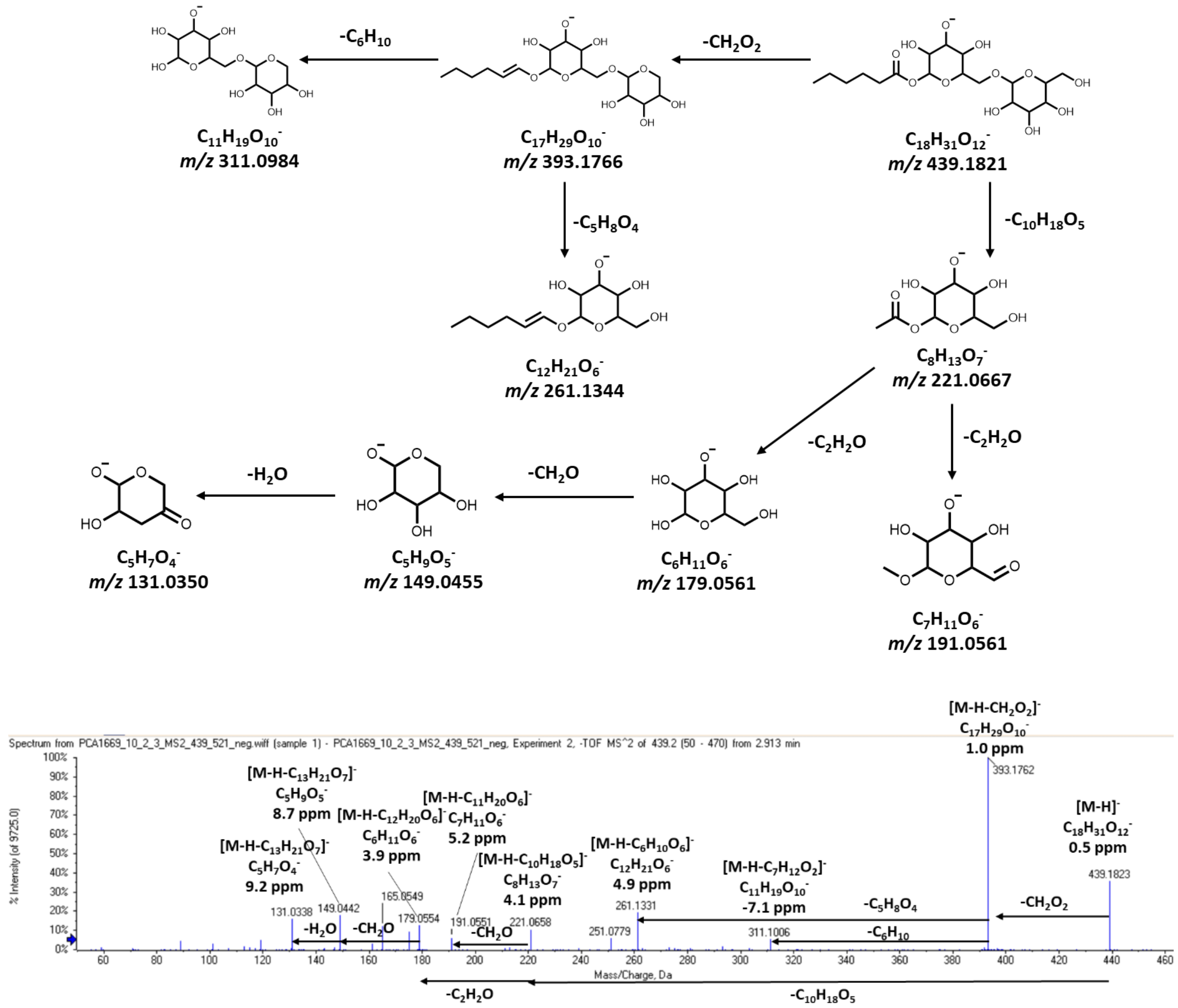

Figure S2-1. Tandem mass spectrum of *m/z* 439.2 at t_R 2.91 min corresponding to 6-O-hexopyranosyl-1-O-hexanoyl-β-D-hexopyranose (**3**). The spectrum was acquired with a hybrid QqTOF mass spectrometer operated in the negative product ion mode with unit Q1 resolution (collision energy 20 eV). Structures shown are suggestions.

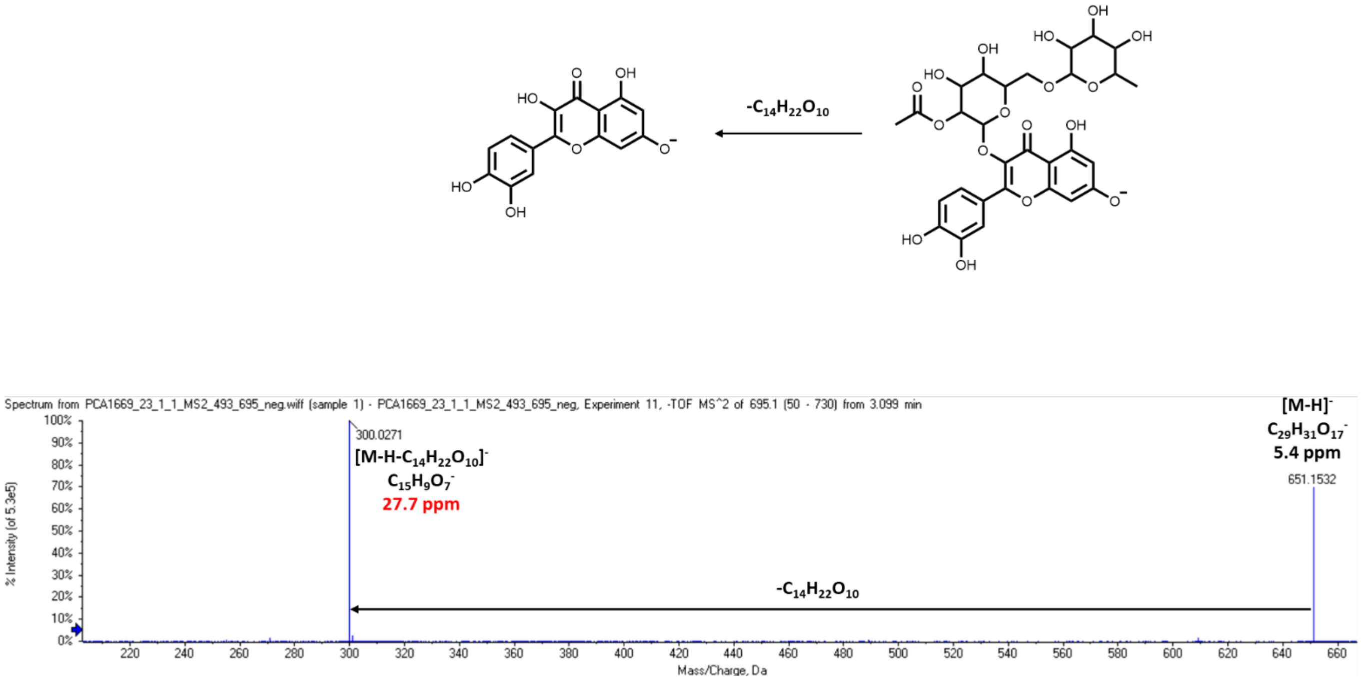

Figure S2-2. Tandem mass spectrum of *m/z* 695.1 at t_R 3.09 min corresponding to quercetin acetyl-hexosyl-deoxy-hexoside (**6**). The spectrum was acquired with a hybrid QqTOF mass spectrometer operated in the negative product ion mode with unit Q1 resolution (collision energy 20 eV). Structures shown are suggestions.

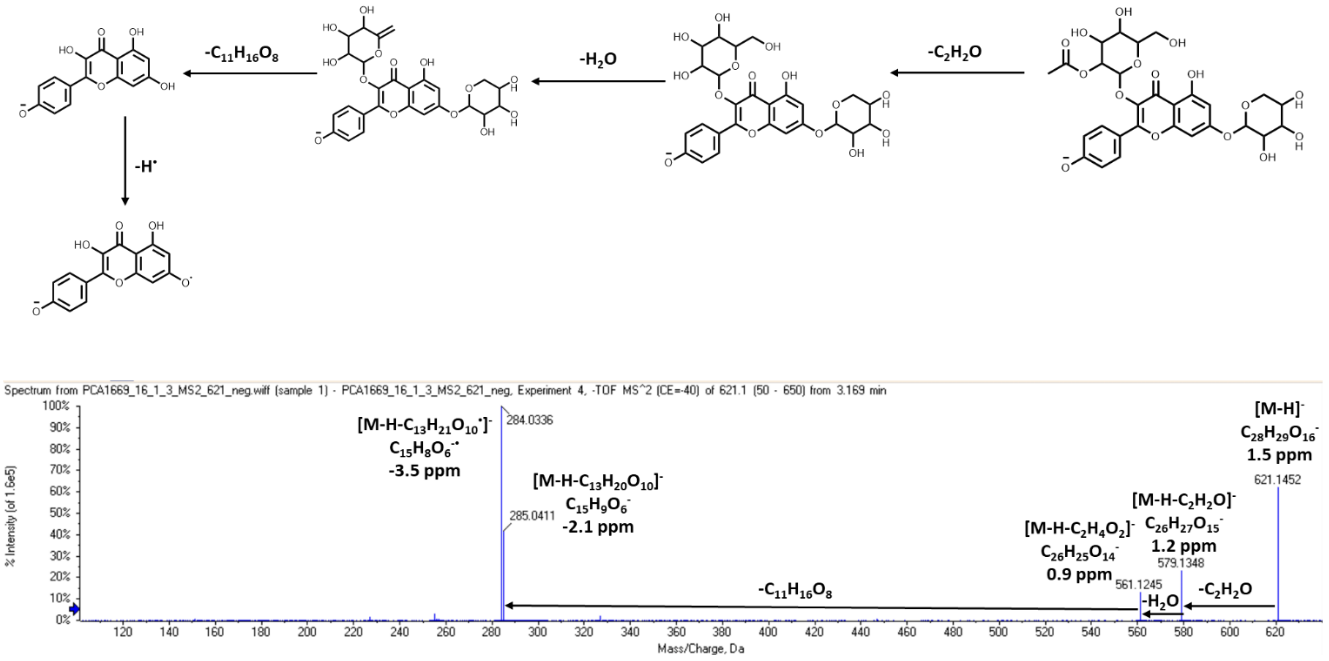

Figure S2-3. Tandem mass spectrum of *m/z* 621.2 at t_R 3.17 min corresponding to kaempferol 3-O-(2''-acetyl)pentopyranoside-7-O-hexopyranoside (**10**). The spectrum was acquired with a hybrid QqTOF mass spectrometer operated in the negative product ion mode with unit Q1 resolution (collision energy 20 eV). Structures shown are suggestions.

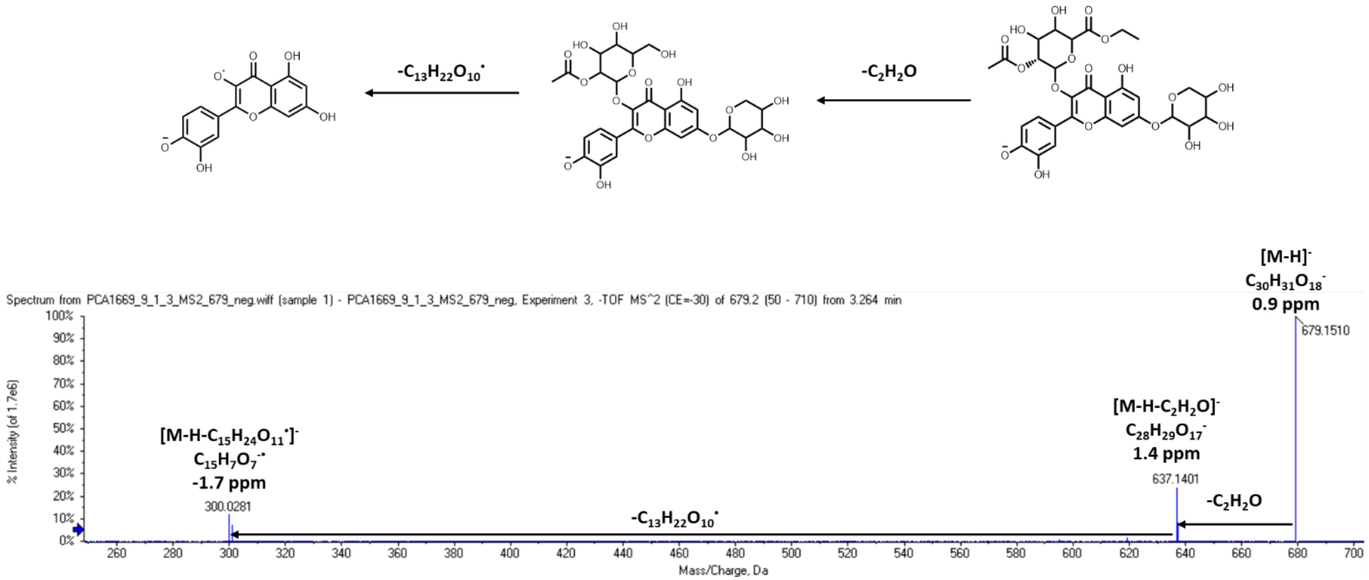

Figure S2-4. Tandem mass spectrum of *m/z* 679.2 at t_R 3.26 min corresponding to quercetin 3-O-(2''-acetyl-6''-ethyl)hexuronide-7-O-pentoside isomer 1 (**12**). The spectrum was acquired with a hybrid QqTOF mass spectrometer operated in the negative product ion mode with unit Q1 resolution (collision energy 20 eV). Structures shown are suggestions.

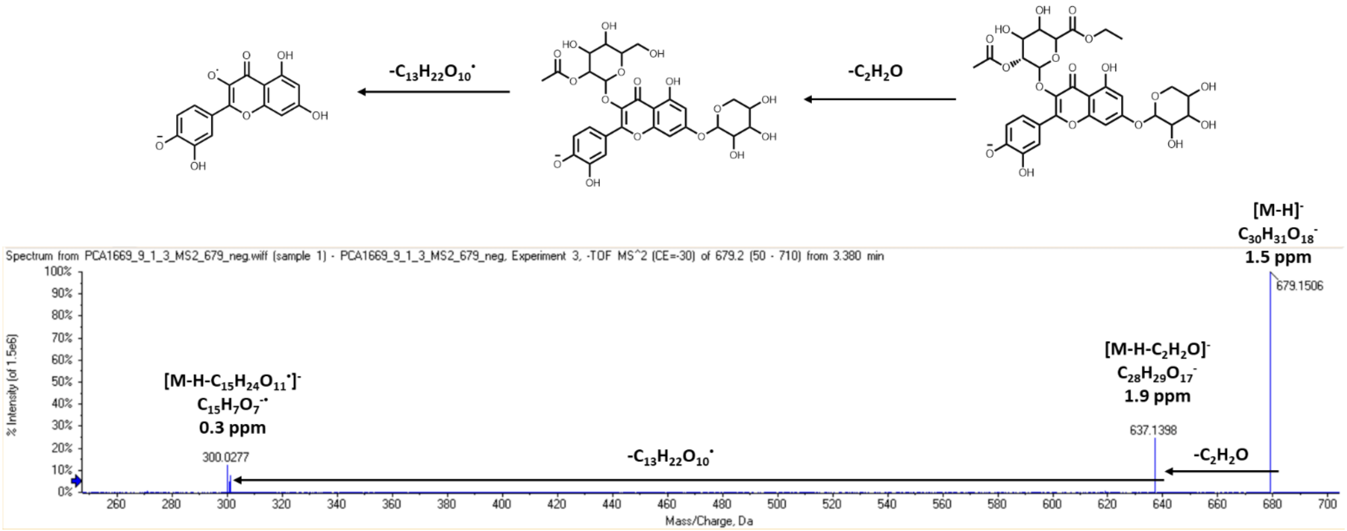

Figure S2-5. Tandem mass spectrum of *m/z* 679.2 at t_R 3.38 min corresponding to quercetin 3-O-(2''-acetyl-6''-ethyl)hexuronide-7-O-pentoside isomer 2 (**15**). The spectrum was acquired with a hybrid QqTOF mass spectrometer operated in the negative product ion mode with unit Q1 resolution (collision energy 20 eV). Structures shown are suggestions.

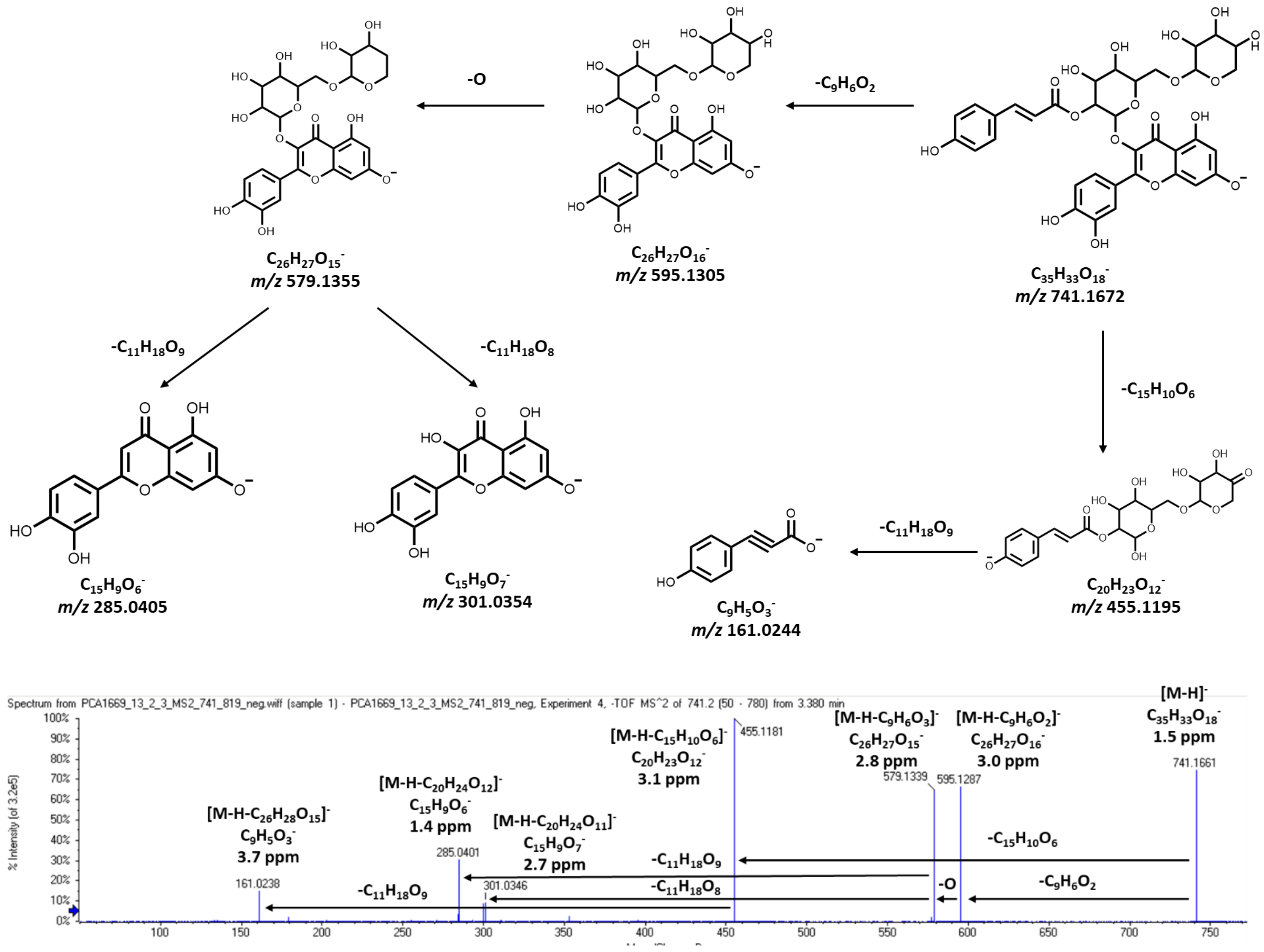

Figure S2-6. Tandem mass spectrum of *m/z* 741.2 at t_R 3.38 min corresponding to quercetin 3-O-hexopyranosyl-(1->6)-[2"-O-p-coumaroyl]-pentopyranoside (**16**). The spectrum was acquired with a hybrid QqTOF mass spectrometer operated in the negative product ion mode with unit Q1 resolution (collision energy 20 eV). Structures shown are suggestions.

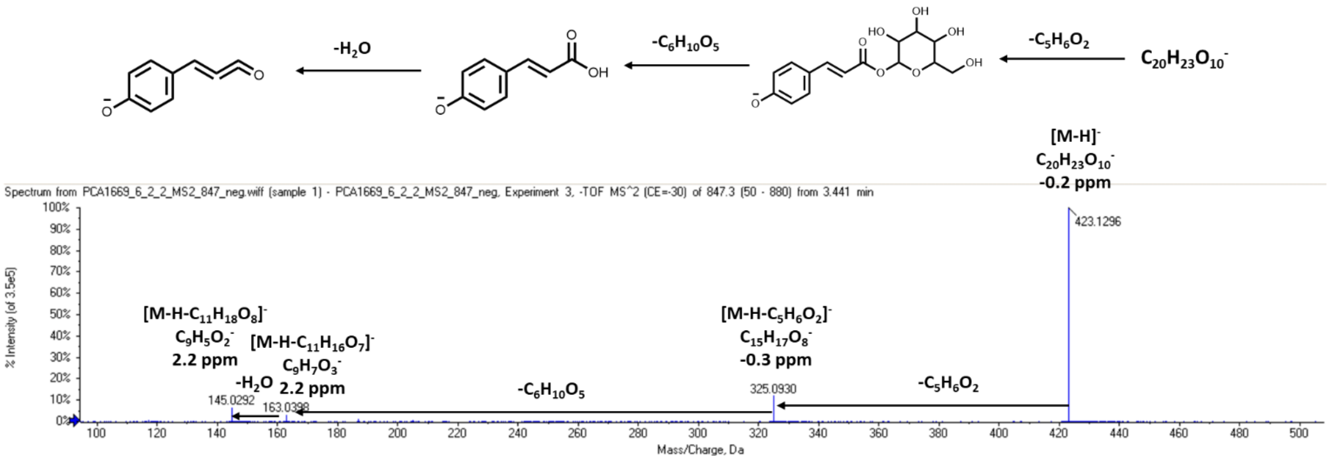

Figure S2-7. Tandem mass spectrum of *m/z* 847.3 at t_R 3.44 min corresponding to coumaroyl hexoside derivatives (**19**). The spectrum was acquired with a hybrid QqTOF mass spectrometer operated in the negative product ion mode with unit Q1 resolution (collision energy 20 eV). Structures shown are suggestions.

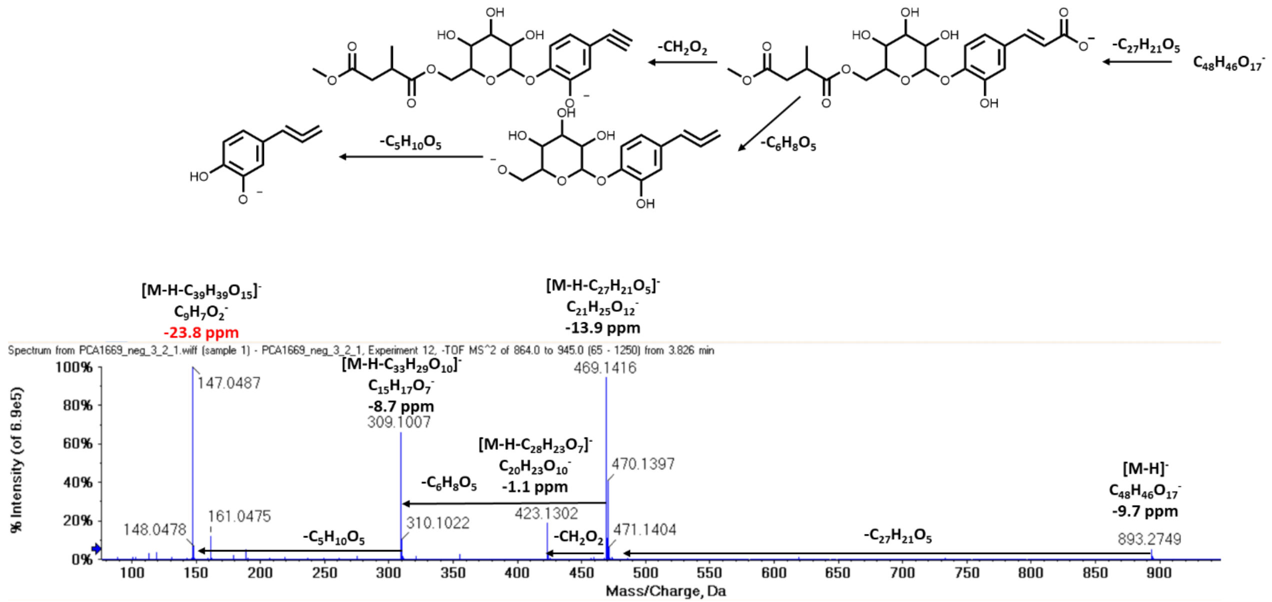

Figure S2-8. Tandem mass spectrum of *m/z* 893.3 at t_R 3.8 min corresponding to 3-[[6-O-[3-(4-hydroxyphenyl)-1-oxoprop-2-en-1-yl]-glucopyranosyl]oxy]-3-methylglutaric acid (**21**). The spectrum was acquired with a hybrid QqTOF mass spectrometer operated in the negative product ion mode with unit Q1 resolution (collision energy 20 eV). Structures shown are suggestions.

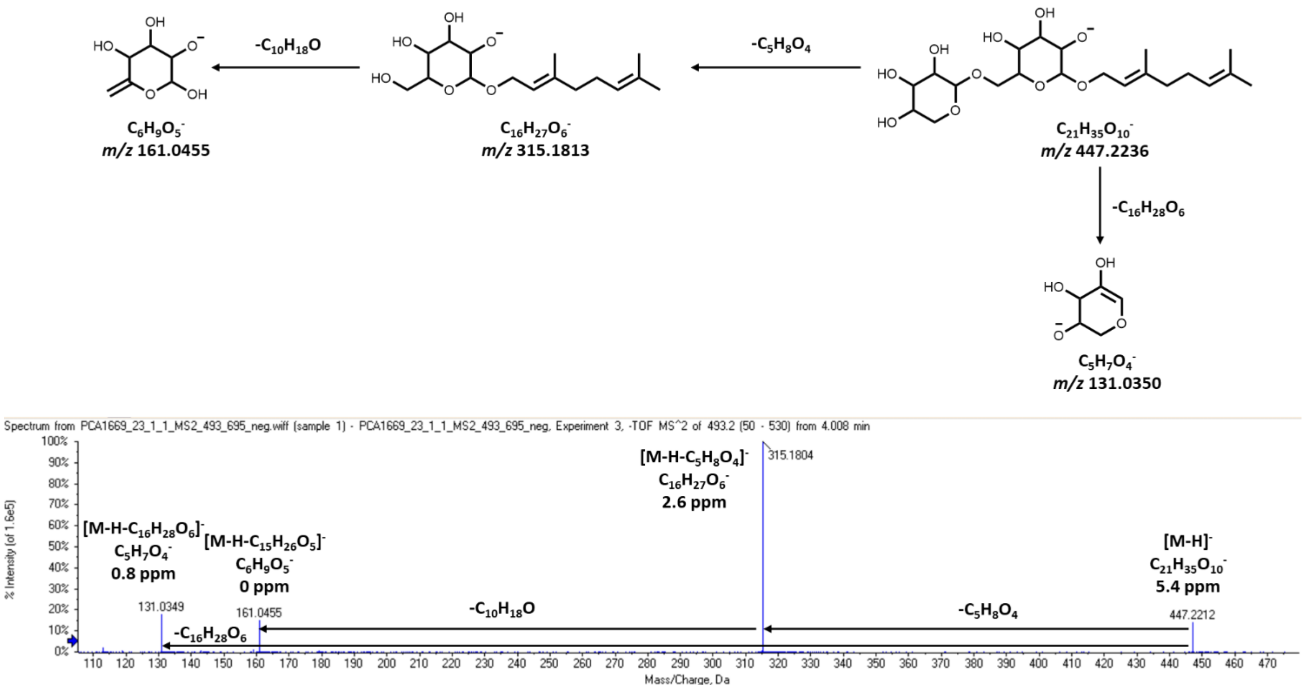

Figure S2-9. Tandem mass spectrum of *m/z* 493.2 at t_R 4.01 min corresponding to geranyl 6-O- pentopyranosyl- hexopyranoside (**23**). The spectrum was acquired with a hybrid QqTOF mass spectrometer operated in the negative product ion mode with unit Q1 resolution (collision energy 20 eV). Structures shown are suggestions.

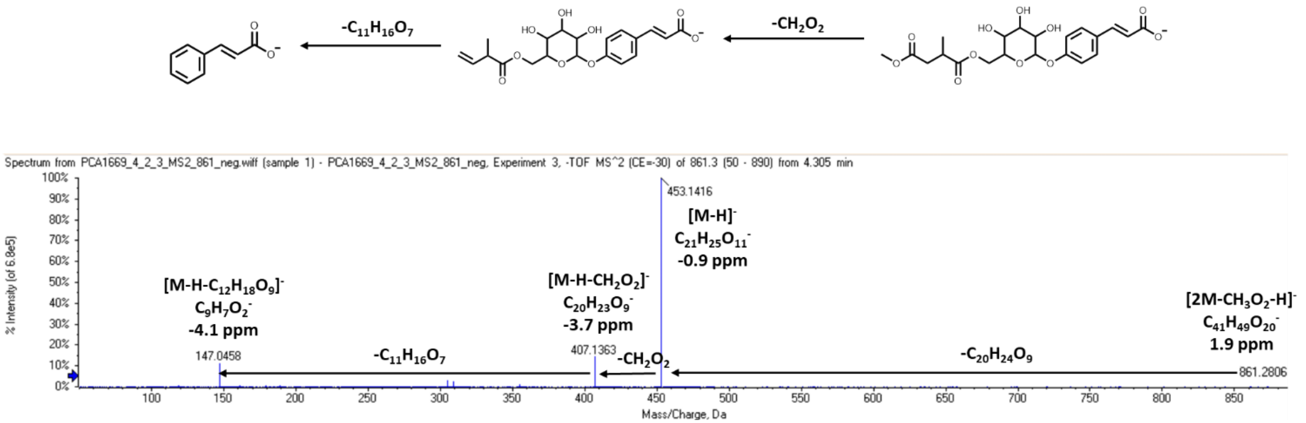

Figure S2-10. Tandem mass spectrum of *m/z* 861.3 at t_R 4.3 min corresponding to argutinoside D (**26**). The spectrum was acquired with a hybrid QqTOF mass spectrometer operated in the negative product ion mode with unit Q1 resolution (collision energy 20 eV). Structures shown are suggestions.

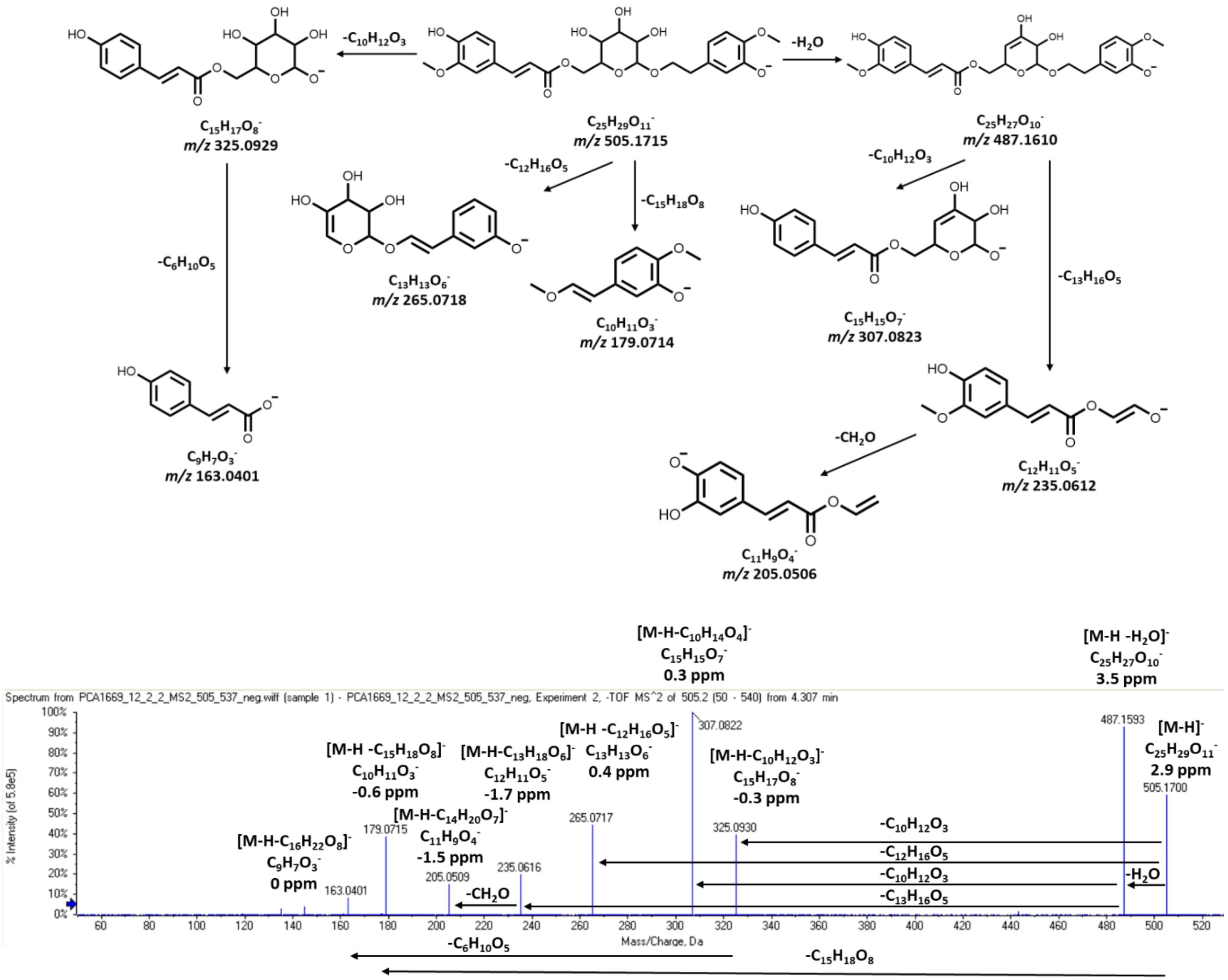

Figure S2-11. Tandem mass spectrum of *m/z* 505.2 at t_R 4.31 min corresponding to 2-(3-hydroxy-4-methoxyphenyl)ethyl 6′-O-feruloyl-hexopyranoside (**27**). The spectrum was acquired with a hybrid QqTOF mass spectrometer operated in the negative product ion mode with unit Q1 resolution (collision energy 20 eV). Structures shown are suggestions.

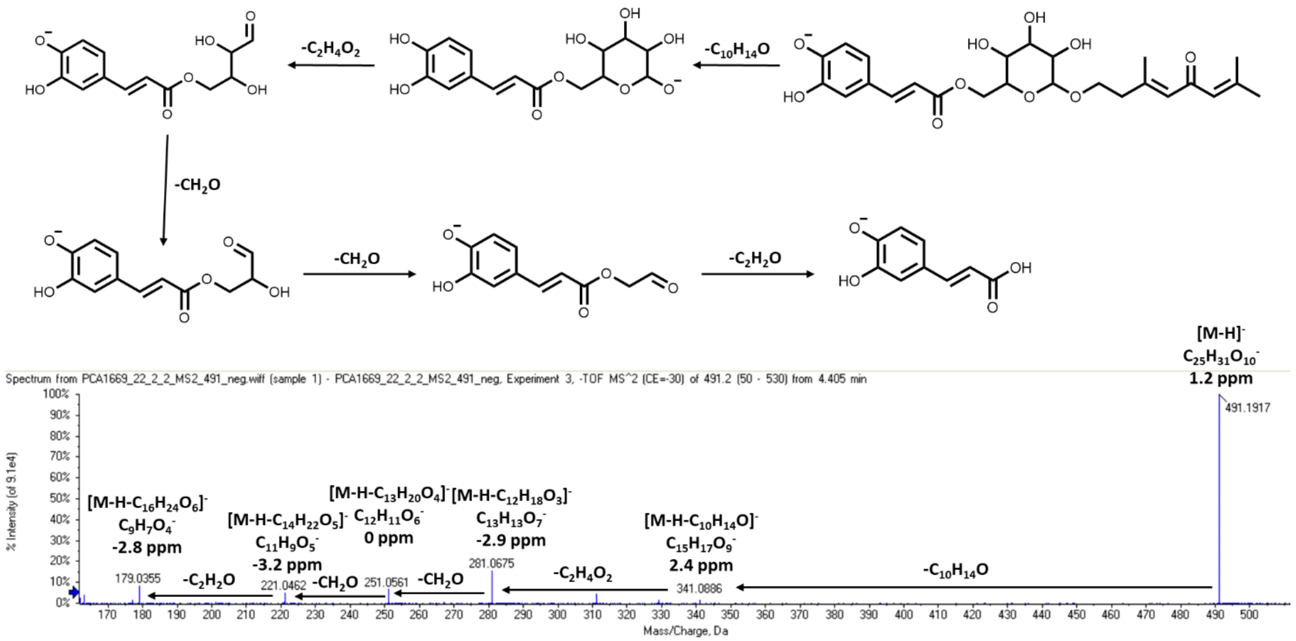

Figure S2-12. Tandem mass spectrum of *m/z* 491.2 at t_R 4.41 min corresponding to 6''-O-cafeoyl-1''-O-hexopyranosy-3,7-dimethyl-3,6-octadien-5-one (**28**). The spectrum was acquired with a hybrid QqTOF mass spectrometer operated in the negative product ion mode with unit Q1 resolution (collision energy 20 eV). Structures shown are suggestions.

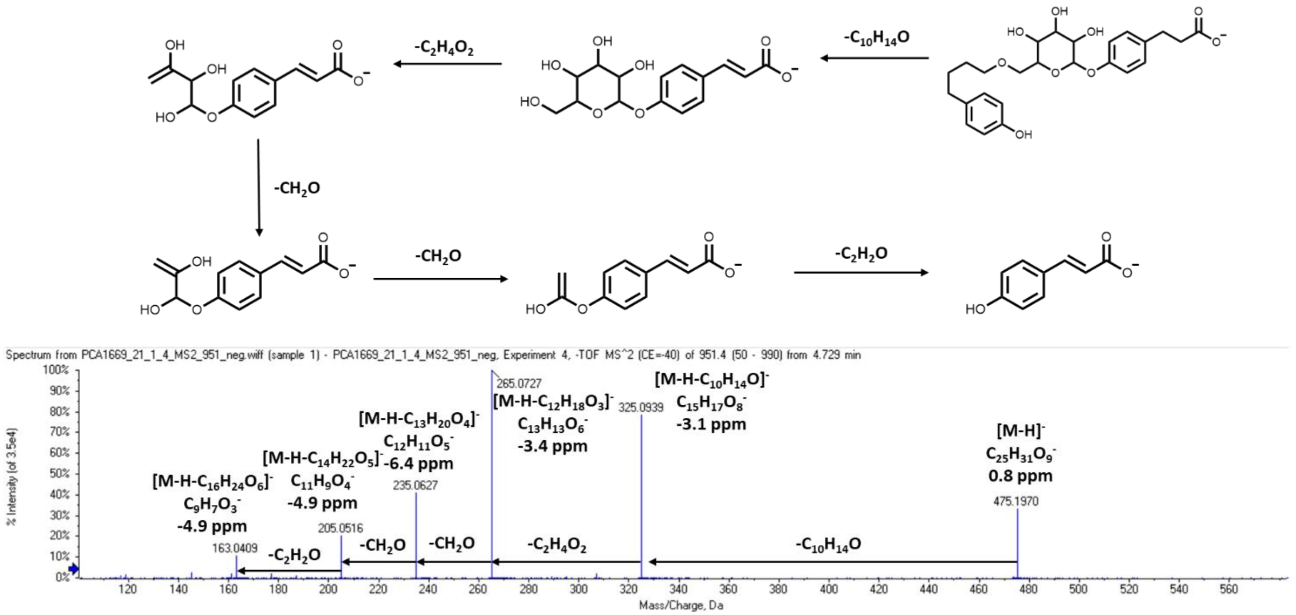

Figure S2-13. Tandem mass spectrum of *m/z* 475.2 at t_R 4.7 min corresponding to 6-O-phenylbytil-1-O-dihydrocoumaroyl-hexoside (**31**). The spectrum was acquired with a hybrid QqTOF mass spectrometer operated in the negative product ion mode with unit Q1 resolution (collision energy 20 eV). Structures shown are suggestions.

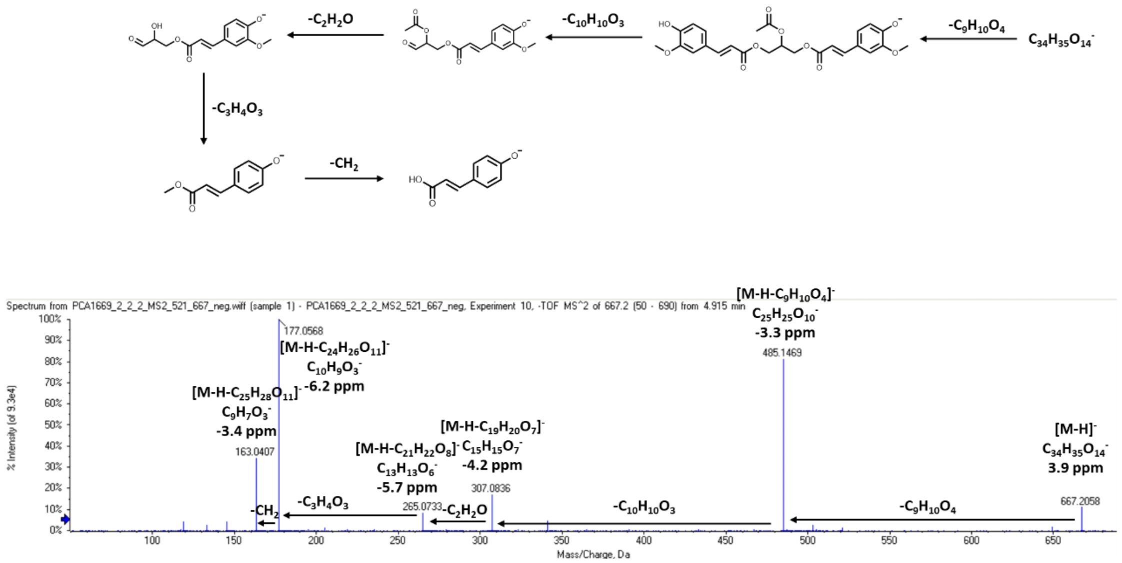

Figure S2-14. Tandem mass spectrum of *m/z* 667.2 at t_R 4.9 min corresponding to unknown derivatives of 2-acetyl-1,3-di-feruloyl-glycerol (**32**). The spectrum was acquired with a hybrid QqTOF mass spectrometer operated in the negative product ion mode with unit Q1 resolution (collision energy 20 eV). Structures shown are suggestions.

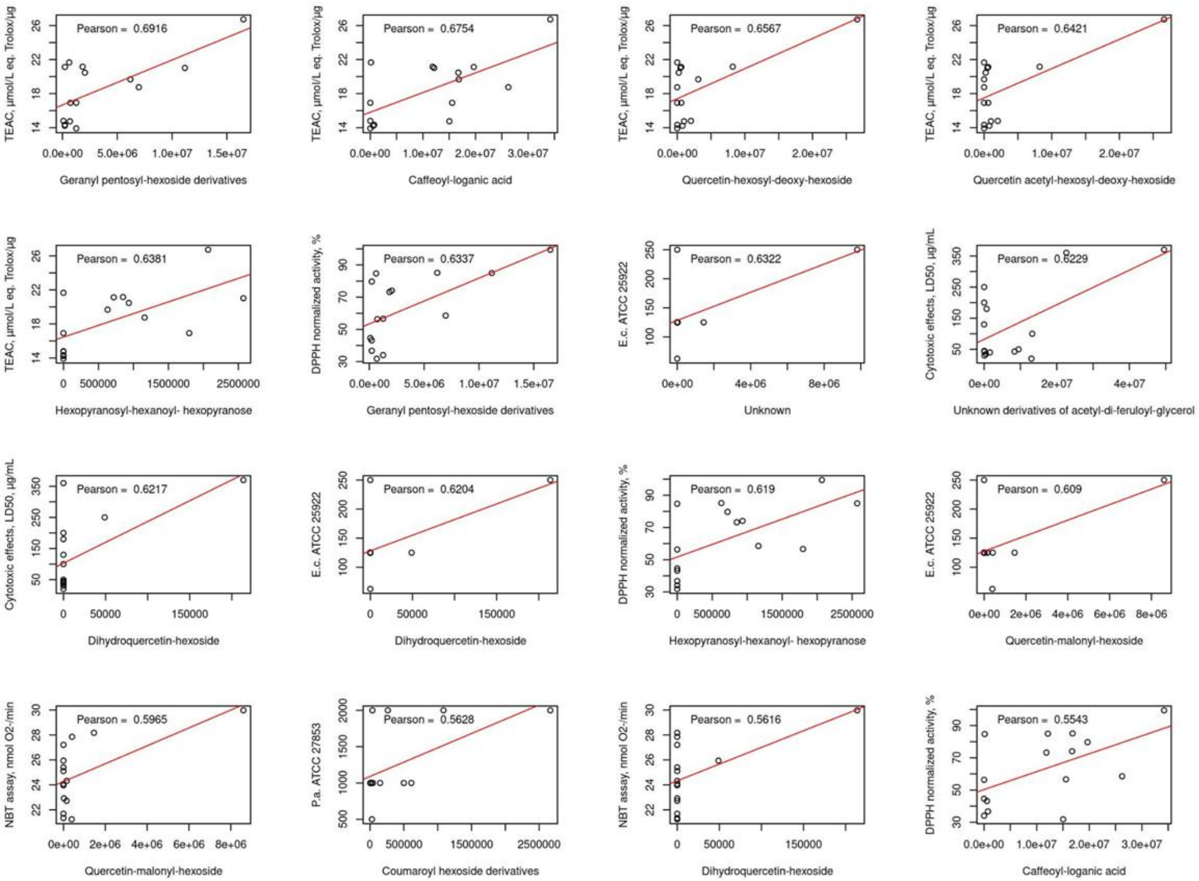

Figure S3. Correlation analysis of the dependence of the investigated types of biological activity on the content of major components of methanol extracts of first-year shoots of the investigated species of the genus *Spireae*. The top-16 activity-metabolite pairs according to the reliable values of the pairwise Pearson correlation coefficient are presented

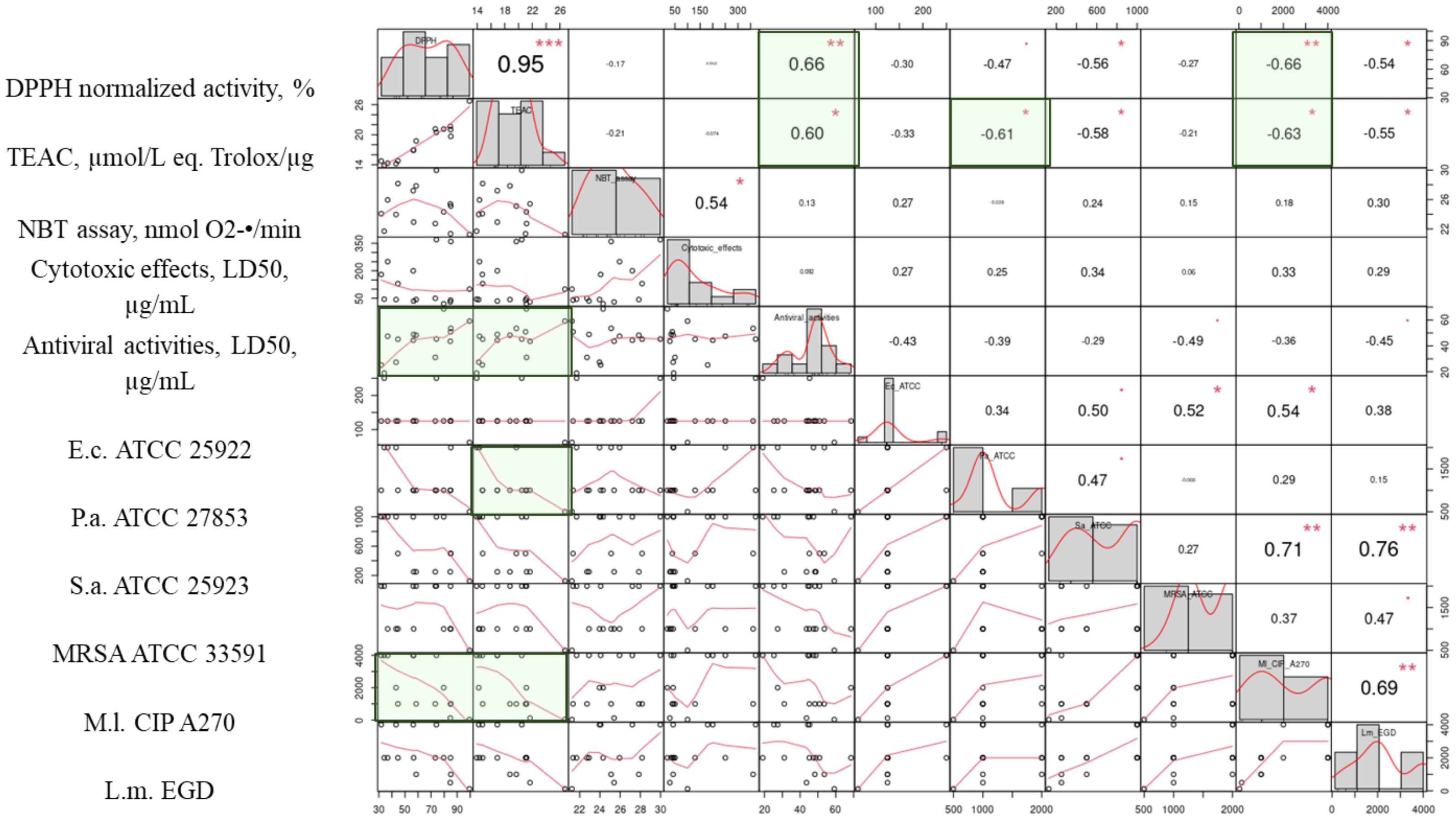

Figure S4. Graph of mutual dependence of manifestation of different types of biological activity of methanolic extracts of first-year shoots of plants of *Spireae* genus selected for the study. The pairs with antioxidant activities, for which the values of Pearson correlation coefficient are statistically significant and exceed 0.6, are marked in green

Annotation of major metabolites in methanolic extracts of first year shoots of fifteen species of plants of the *Spirea* genus by reversed phase ultra-high-performance liquid chromatography—tandem mass spectrometry (RP-UHPLC-QqTOF-MS/MS)

Among the annotated compounds, twelve compounds were featured with a common fragmentation pattern, dominated with the signals at *m/z* 300.0281 and characteristic for quercetin derivatives or signal at *m/z* 301.0346 which correspondent to the fragment of the quercetin aglycon.

Thus, compound **4** (*m/z* 595.1358, t_R 2.9 min corresponding to the elemental composition $C_{26}H_{27}O_{16}^-$, 9.0 ppm (here and further practical mass-to-charge ratios are presented, theoretical ones could be found in Table 1)) was annotated as quercetin-hexosyl pentoside due to the presence of the characteristic *m/z* 300.0281 corresponding to quercetin resulting from the neutral loss of the hexosyl-pentoside moiety [M-H-295] and appeared to be a characteristic major metabolite of *S. salicifolia f. alpestris* (Figure S1-3). Further, compound **5** (*m/z* 609.1474, t_R 3.0 min corresponding to the elemental composition $C_{27}H_{29}O_{16}^-$, -2.1 ppm) was annotated as quercetin-hexosyl-deoxyhexoside by the presence of a characteristic ion *m/z* 300.0262 formed by neutral loss of hexosyl-deoxy-hexoside moiety [M-H-309] (Figure S1-4), and compound **6** (*m/z* 695.1402, t_R 3.1 min corresponding to the elemental formula $C_{30}H_{31}O_{19}^-$, 9.1 ppm) was annotated as acetyl-quercetin-hexosyl-deoxyhexoside due to the presence of the characteristic loss of the acetyl-hexosyl-deoxy-hexoside moiety [M-H-351] resulting in the fragment ion at *m/z* 300.0271 (quercetin aglycone) (Figure S2-2). These two conjugates were characteristic for the methanol extract of *S. humilis*. The compound **8** (*m/z* 787.1808, t_R 3.1 min corresponding to the elemental formula $C_{40}H_{47}O_{22}^-$, 10.4 ppm) was annotated as quercetin-trihexoside in *S. chamaedryfolia* extract by the presence of sequential neutral loss of one and two hexose residues (Figure S1-6), whereas the compound **9** (*m/z* 927.1833, t_R 3.2 min corresponding to the elemental formula $C_{42}H_{39}O_{24}^-$, 0.4 ppm) was annotated as quercetin-hexoside due to the presence of the characteristic loss of the hexoside moiety [M-H-163] in the

extracts prepared from the shoots of *S. aquilegifolia, S. crenata, S. hypericifolia, S. media, S. pubescens, S. sericea, S. trilobata* (Figure S1-7). Also, compound **12** (*m/z* 679.1510, t_R 3.3 min corresponding to the elemental composition $C_{30}H_{31}O_{18}^-$, 0.9 ppm) was annotated as quercetin acetyl-ethyl-hexuronide-*O*-pentoside isomer 1 in the extracts of *S. media* and *S. sericea* (Figure S2-4), whereas compound **14** (*m/z* 1099.1840, t_R 3.4 min corresponding to the elemental formula $C_{48}H_{43}O_{30}^-$, 1.5 ppm) was annotated as quercetin-malonyl-hexoside by the presence of sequential neutral loss of malonic acid [M-H-86] and hexoside [M-H-163] moieties and detected in the extract of only one species, namely *S. hypericifolia* (Figure S1-10). In turn, compound **15** (*m/z* 679.1506, t_R 3.38 min corresponding to the elemental formula $C_{30}H_{31}O_{18}^-$, 1.5 ppm) was annotated as quercetin acetyl-ethyl-hexuronide-*O*-pentoside isomer 2 and characteristic for *S. media* extract (Figure S2-5) and compound **18** (*m/z* 771.1816, t_R 3.43 min corresponding to the elemental composition $C_{36}H_{35}O_{19}^-$, -4.9 ppm), which appeared to be characteristic for two species - *S. schamaedryfolia* and *S. crenata,* and was annotated as quercetin-coumaroyl-dihexoside (Figure S1-12). All the above listed compounds were annotated as the quercetine derivative by the presence of the characteristic fragment at *m/z* 300.02 in their spectra, which was clear visible both in the SWATH and product ion spectra (Table 1, Figures S1-3, S1-4, S1-6, S1-7, S1-10, S1-12, S2-2, S2-4, S2-5).

Finally, the three major compounds of this group were annotated by the presence in their spectra a signal at *m/z* 301.0346 which correspondent to the fragment of the quercetin aglycon. This signal was present in the tandem mass spectra of the compound **13** (*m/z* 757.1708, t_R 3.3 min corresponding to the elemental formula $C_{35}H_{33}O_{19}^-$, -10.3 ppm), which was characteristic for the *S. salicifolia* and *S. salicifolia f. alpestris* extracts and was annotated as caffeoyl pentoside-quercetin hexoside (Figure S1-9). The same signal was found in the MS/MS spectra of the compound **16** (*m/z* 741.1661, t_R 3.4 min corresponding to the elemental composition $C_{35}H_{33}O_{18}^-$, 1.5 ppm), which was annotated as quercetin hexopyranosyl-*O*-*p*-coumaroyl-pentopyranoside and was characterized in the methanolic extracts of *S.sericea* and

S. ussuriensis shoots (Figure S2-6). By the same fragment was recognized the compound **17** (*m/z* 867.1605, t_R 3.4 min corresponding to the elemental formula $C_{40}H_{35}O_{22}^-$, 2.3 ppm), annotated as quercetin-pentoside and found in the extracts of *S.aquilegifolia* (Figure S1-11). The compound **2** (*m/z* 465.0983, $\mathbf{t_R}$ 2.8 min corresponding to the elemental formula $C_{21}H_{21}O_{12}^-$, 10.8 ppm) was the last compound representing the group the quercetin derivatives. It was annotated as dihydroquercetin-hexoside and detected in the extracts of two species of the genus *Spiraea* (*S. media, S.sericea*), annotated by the presence of a dehydroxy-quercetin fragment ion with *m/z* 285.0373 in the corresponding target MS/MS spectrum (Figure S1-2).

The second group of the major compounds was represented also by flavonoids, namely - kaempferol derivatives. Annotation of these derivatives relied on the characteristic signal at *m/z* 285.0413 present in all corresponding MS/MS spectra and corresponding to the fragment ion of the kaempferol aglycon. Among the compounds belonging to this group were the compounds **10** and **11**. The annotation of compound **10** (*m/z* 621.1452, t_R 3.2 min, $C_{28}H_{29}O_{16}^-$, 1.5 ppm), characteristic for the methanolic extracts prepared from the first year shoots of *S. betulifolia*, was confirmed by the presence of fragment signals corresponding to the sequential neutral losses of acetyl, pentose and hexose. Based on manual interpretation of the tandem mass spectrum, compound **10** was annotated as kaempferol acetyl-pentopyranoside-*O*-hexopyranoside (Figure S2-3). Compound **11** (*m/z* 1127.2890, t_R 3.2 min, $C_{52}H_{55}O_{28}^-$, -0.2 ppm), characteristic for *S. elegans*, was annotated by the presence of the signals corresponding to the neutral losses of the pentosyl moiety and the dehydroxy-hexoside. Based on this fragment pattern, this compound was annotated as kaempferol-pentosyl-deoxyhexoside (Figure S1-8).

The remaining 15 compounds were assigned to the class of hydroxycinnamic acids - derivatives of caffeic, *p*-coumaric, ferulic acids. Based on the presence of caffeic acid fragment ion (*m/z* 179.0355) or neutral loss of caffeic acid in the fragmentation patterns of the studied compounds, major constituents **1, 7, 22** and **28** of the spirea extracts were assigned to the group of caffeic acid derivatives. The elemental composition of compounds **1** (*m/z* 707.1816, t_R 2.2

min, $C_{32}H_{35}O_{18}^-$, 4.5 ppm), characteristic for all studied extracts, **7** (*m/z* 879.2546, t_R 3.1 min, $C_{40}H_{47}O_{22}^-$, 2.1 ppm), found in *S. elegans* extract, **22** (*m/z* 537.1627, t_R 3.9 min, $C_{25}H_{29}O_{13}^-$, -2.4 ppm), characteristic for *S. aquilegifolia*, and **28** (*m/z* 491.1917, t_R 4.4 min, $C_{25}H_{31}O_{10}^-$, 1.2 ppm), characteristic for *S. salicifolia* and *S. salicifolia f. alpestris*, was determined from ESI-HR-MS data (Table 1). Based on these data, compound **1** was annotated as caffeoyl-quinic acid, compound **7 -** as caffeoyl-hydroxy-methylbutyryl-pentoside, compound **22** as caffeoyl-loganic acid, and compound **28** was assigned as caffeoyl-hexopyranosy-dimethyl-octadienone (Figure S1-1, S1-5, S1-13, S2-12).

The tandem mass spectra of the compounds **19, 24, 26, 29 and 31** showed a characteristic signal *m/z* 163.0409 corresponding to the fragment ion of coumaric acid. Therefore, these major components could be assigned to the group of coumaric acid derivatives (*m/z* 847.2691, t_R 3.4 min, $C_{40}H_{47}O_{20}^-$, 2.9 ppm), found in extracts of *S. elegans*, **24** (*m/z* 1043.3410, t_R 4.2 min, $C_{50}H_{59}O_{24}^-$, -0.9 ppm), fairly widely distributed in extracts of the studied species, **26** (*m/z* 861.2806, t_R 4.30 min, $C_{41}H_{49}O_{20}^-$, 1.9 ppm), characteristic for *S. crenata* and *S. ussuriensis*, **29** (*m/z* 521.1639, t_R 4.42 min, $C_{25}H_{29}O_{12}^-$, 4.8 ppm), characteristic for *S. aquilegifolia, S. flexuosa, S. hypericifolia, S. pubescens, S. betulifolia* and **31** (*m/z* 475.1970, t_R 4.7 min, $C_{25}H_{31}O_9^-$, 0.8 ppm), detectable in *S. salicifolia* extract, could be annotated based on the ESI-HR-MS and MS/MS data (Table 1). all the analytes, with the exception of compound **26**, The MS/MS spectra of compound **26** contained a characteristic fragment of dehydroxy-coumaric acid (*m/z* 147.0458). Because of this, this natural product was also assigned to the group of coumaric acid derivatives. The presence of the characteristic fragment *m/z* 325.0930 and neutral loss of 163 u allowed annotation of compound **19** as a *p*-coumaroyl-hexoside derivative, compound **24** as a *p*-coumaroyl-loganic acid isomer 1, compound **26** as a *p*-coumaroyl hexoside derivative, compound **29** as a *p*-coumaroyl-loganic acid isomer 2, and compound **31** as a phenylbytil-*O*-dihydrocoumaroyl-hexoside (Figures S2-7, S1-14, S2-10, S1-15, S2-13).

The elemental compositions of compounds **27** (*m/z* 505.1700, t_R 4.3 min, $C_{25}H_{29}O_{11}^-$, 2.9 ppm) and **32** (*m/z* 667.2029, t_R 4.9 min, $C_{34}H_{35}O_{14}^-$, 3.9 ppm) were predicted as $C_{25}H_{29}O_{11}^-$ and $C_{34}H_{35}O_{14}^-$, respectively on the basis of ESI-HR-MS data (Table 1). Further, based on the presence of the signals corresponding to the neutral loss of 270 u corresponding to the cleavage of ferulic acid moiety in the tandem mass spectra, these major constituents of the *S. trilobata, S.aquilegifolia, S. crenata, S. hypericifolia* extracts were assigned to the group of ferulic acid derivatives due to the. Thus, the tandem mass spectrum of compound **27** revealed a fragment at *m/z* 235.0616 corresponding to ferulic acid hydroxy-vinyl moiety (neutral loss of 270 u). Thus, compound **27** could be annotated as (hydroxy-methoxyphenyl)ethyl-*O*-feruloyl-hexopyranoside (Figure S2-11). The tandem mass spectrum of compound **32** revealed a fragment at *m/z* 265.0733 corresponding to hydroxy-oxopropyl ester of ferulic acid. Thus, based on the results of manual interpretation of the tandem mass spectrum, compound **32** was annotated as an unknown derivative of acetyl-di-feruloyl-glycerol (Figure S2-14).

Compound **3** (*m/z* 439.1823, t_R 2.8 min, $C_{21}H_{21}O_{12}^-$, 10.8 ppm), characteristic for the methanolic extracts prepared from the first year shoots of *S.pubescens*, was annotated by the presence of the $[M-H]^-$ signal at *m/z* 439.1823 in the mass spectrum, along with the fragment ion signals at *m/z* 221.0667 and at *m/z* 179.0561 (corresponding to the acetyl hexose and hexose fragments, respectively) in the tandem mass spectrum. Based on this fragmentation pattern, compound **3** was annotated as *O*-hexopyranosyl-*O*-hexanoyl-hexopyranose (Figure S2-1).

Compound **21** (*m/z* 893.2781, t_R 3.8 min, $C_{41}H_{40}O_{22}^-$, -6.7 ppm), characteristic for the methanolic extracts prepared from the first year shoots of *S. chamaedryfolia, S. crenata* and *S. ussuriensis*, was annotated by the presence of the $[M-H]^-$ signal at *m/z* 469.1416 in the mass spectrum, along with the fragment ion at *m/z* 309.1007 in the tandem mass spectrum obtained by the neutral loss of glutaric acid and carbon monoxide. Based on this fragmentation pattern, compound **21** was annotated as (hydroxyphenyl)-oxopropenyl-glucopyranosyloxy-methylglutaric acid (Figure S2-8).

Compound **23** (*m/z* 493.2212, t_R 4.2 min, $C_{29}H_{33}O_7^-$, 3.9 ppm), characteristic for the methanolic extracts of *S. humilis*, was annotated by the presence of a fragment signal at *m/z* 447.2212 (characteristic for geranyl pentosyl-hexoside) and a fragment at *m/z* 315.1804 (characteristic for geranyl-hexoside) in the tandem mass spectrum. Based on this fragmentation pattern, compound **23** could be annotated as geranyl pentosyl-hexoside derivatives (Figure S2-9).
